# A major role for RCAN1 in atherosclerosis progression

**DOI:** 10.1002/emmm.201302842

**Published:** 2013-10-15

**Authors:** Nerea Méndez-Barbero, Vanesa Esteban, Silvia Villahoz, Amelia Escolano, Katia Urso, Arantzazu Alfranca, Cristina Rodríguez, Susana A Sánchez, Tsuyoshi Osawa, Vicente Andrés, José Martínez-González, Takashi Minami, Juan Miguel Redondo, Miguel R Campanero

**Affiliations:** 1 Department of Vascular Biology and Inflammation, Centro Nacional de Investigaciones CardiovascularesMadrid, Spain; 2 Human Genetics Department, Institute for Rare Diseases Research, Carlos III Health InstituteMadrid, Spain; 3 Centro de Investigación Cardiovascular (CSIC-ICCC)IIB Sant Pau, Barcelona, Spain; 4 Microscopy and Dynamic Imaging Unit, Centro Nacional de Investigaciones CardiovascularesMadrid, Spain; 5 Division of Vascular Biology, The Research Center for Advanced Science and Technology (RCAST), The University of TokyoTokyo, Japan; 6 Department of Epidemiology, Atherothrombosis and Imaging, Centro Nacional de Investigaciones CardiovascularesMadrid, Spain; 7 Department of Cancer Biology, Instituto de Investigaciones Biomedicas Alberto SolsCSIC-UAM, Madrid, Spain

**Keywords:** atherosclerosis, hypercholesterolemia, inflammation, macrophage, RCAN1

## Abstract

Atherosclerosis is a complex inflammatory disease involving extensive vascular vessel remodelling and migration of vascular cells. As RCAN1 is implicated in cell migration, we investigated its contribution to atherosclerosis. We show RCAN1 induction in atherosclerotic human and mouse tissues. Rcan1 was expressed in lesional macrophages, endothelial cells and vascular smooth muscle cells and was induced by treatment of these cells with oxidized LDLs (oxLDLs). Rcan1 regulates CD36 expression and its genetic inactivation reduced atherosclerosis extension and severity in *Apoe*^*−/−*^ mice. This effect was mechanistically linked to diminished oxLDL uptake, resistance to oxLDL-mediated inhibition of macrophage migration and increased lesional IL-10 and mannose receptor expression. Moreover, *Apoe*^*−/−*^*Rcan1*^*−/−*^ macrophages expressed higher-than-*Apoe*^*−/−*^ levels of anti-inflammatory markers. We previously showed that Rcan1 mediates aneurysm development and that its expression is not required in haematopoietic cells for this process. However, transplantation of *Apoe*^*−/−*^*Rcan1*^*−/−*^ bone-marrow (BM) cells into *Apoe*^*−/−*^ recipients confers atherosclerosis resistance. Our data define a major role for haematopoietic Rcan1 in atherosclerosis and suggest that therapies aimed at inhibiting RCAN1 expression or function might significantly reduce atherosclerosis burden.

## INTRODUCTION

Atherosclerosis, the underlying cause of myocardial infarction, stroke and peripheral vascular disease, is the major cause of morbidity and mortality in the developed world. The initial steps of atherosclerosis are characterized by the subendothelial accumulation of apolipoprotein B-containing low-density lipoproteins (LDLs) in the artery wall. The oxidative modification of these lipoproteins [oxidized LDL (oxLDL)] triggers the activation of the vascular endothelium and drives an influx of monocytes to the vascular intima, where they differentiate into macrophages and phagocytose oxLDL (Hansson & Hermansson, 2011). Although in other contexts macrophages egress from the inflammation site after engulfing unwanted material, in an atherosclerotic plaque the loading of oxLDL into macrophages shifts them to a more sessile, foam-cell phenotype, and these foam cells do not leave the lesion after clearing the lipids (Angeli et al, 2004; Randolph, 2008). The trapping of cholesterol-engorged foam cells causes the plaque to expand through the recruitment of additional leukocytes and vascular smooth muscle cells (VSMCs). As these lesions mature they continue to accumulate extracellular lipids, and the central core of the mature plaque becomes necrotic. Rupture of plaques produce acute coronary syndromes, unstable angina, myocardial infarction and sudden death (Libby, 2002).

Monocytes/macrophages are a relatively heterogeneous population, and the existence of at least two broad classes of macrophage phenotype has been proposed: proinflammatory macrophages (classically activated or M1) and those involved in resolution and repair (alternatively activated or M2) (Gordon & Taylor, 2005). M2 macrophages produce low levels of pro-inflammatory cytokines but high levels of arginase1 (Arg1), mannose receptor (Mrc1 or CD206) and IL10, and have a higher phagocytic capacity and a lower antigen presentation capacity than M1 macrophages (Gordon & Taylor, 2005). The existence of other macrophage phenotypes has been proposed that fit neither the classical nor the alternative activation pattern (Mosser & Edwards, 2008). The heterogeneity of atherosclerotic plaque macrophages has been recognized for many years and several types of macrophages have been found in atherosclerotic lesions (Bouhlel et al, 2007; Khallou-Laschet et al, 2010).

Regulator of calcineurin 1 (*RCAN1*) belongs to a family of endogenous regulators of calcineurin activity (RCAN; previously known as DSCR/MCIP/calcipressin/Adapt78 in mammals) (Davies et al, 2007). The RCAN1 protein is highly conserved (Davies et al, 2007), displaying 96% identity between human and mouse (Strippoli et al, 2000). The human and mouse *RCAN1* genes are expressed as two isoforms, RCAN1-1 and RCAN1-4, that differ at their N terminus as a consequence of alternative promoter usage and first exon usage (Davies et al, 2007; Fuentes et al, 1997). RCAN1-1 and RCAN1-4 have different expression patterns and different regulation mechanisms control their expression. While *RCAN1-1* seems to be constitutively expressed in most tissues, transcription of the *RCAN1-4* variant is induced *de novo* by several stimuli that activate the calcineurin-NFAT pathway (Cano et al, 2005; Crawford et al, 1997; Ermak et al, 2002; Esteban et al, 2011; Minami et al, 2004; Wang et al, 2002; Yang et al, 2000). RCAN1 has been implicated in important physiological and pathological processes, including tumour growth and angiogenesis, sepsis, cardiac hypertrophy, mast-cell function, T-cell survival, and synaptic plasticity and memory (Baek et al, 2009; Harris et al, 2005; Hoeffer et al, 2007; Ryeom et al, 2008; Yang et al, 2009). Rcan1 additionally plays an essential role in the migration of VSMCs in response to angiotensin II stimulation; moreover, *Rcan1* genetic ablation in the mouse confers resistance to abdominal aortic aneurysm and to neointima formation in a restenosis model (Esteban et al, 2011). *Rcan1* in endothelial cells inhibits VEGF-induced migration and *in vitro* tube formation (Iizuka et al, 2004; Minami et al, 2004). In contrast, *Rcan1* knockdown in cancer cell lines increases motility while its forced expression reduces their motility and CN activity (Espinosa et al, 2009). Rcan1 thus appears to have opposite roles in cell migration in different settings. Since most studies have involved the simultaneous inactivation of *Rcan1-1* and *Rcan1-4* or have examined the effect of over-expressing or knocking-down only one of these isoforms, it has not yet been possible to ascribe specific roles to one Rcan1 isoform and not the other.

Here, we investigated the contribution of Rcan1 to atherosclerosis development. We show that RCAN1 is induced in human and mouse atherosclerotic tissues and, using a mouse model of atherosclerosis and bone-marrow (BM) transplantation assays, we demonstrate that Rcan1 in haematopoietic cells promotes atherosclerosis. We also present evidence that the pro-atherogenic action of Rcan1 is mediated by oxLDL-uptake and that macrophage polarization and trapping are central to its pro-atherogenic role.

## RESULTS

### RCAN1-4 is upregulated in human and mouse atherosclerosis lesions

**Figure 1:**
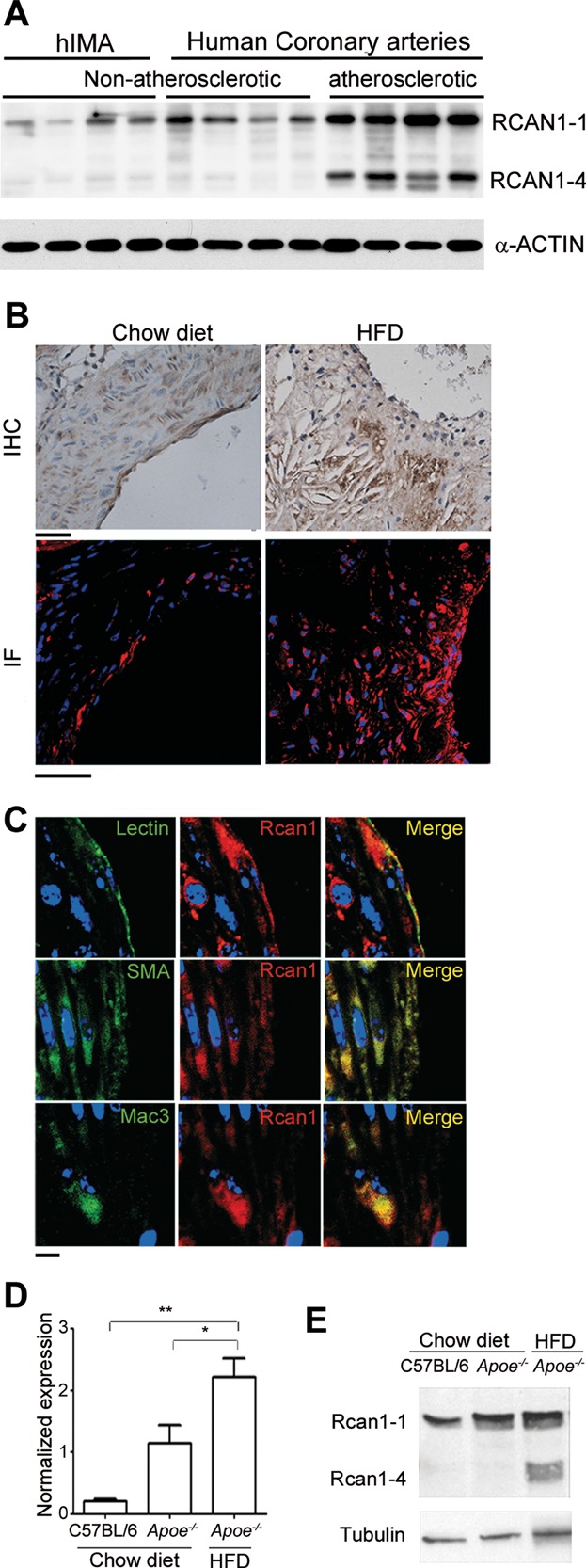
RCAN1-4 is induced in atherosclerotic human and mouse arteries

To assess RCAN1 expression in human atherosclerotic lesions, we compared human atherosclerotic coronary arteries with non-atherosclerotic coronary arteries and internal mammary arteries, a vessel that does not develop atherosclerosis. RCAN1-4 protein expression was markedly higher in atherosclerotic vessels than in non-atherosclerotic coronary arteries and internal mammary arteries (Fig 1A). Although RCAN1-1 expression is usually constitutive, its level also appeared to be higher in atherosclerotic arteries, but the difference was less marked than for RCAN1-4 (Fig 1A). The protein expression differences were accompanied by correspondingly higher expression of *RCAN1-1* and *RCAN1-4* mRNA in atherosclerotic arteries (Fig S1 of Supporting Information).

1. Immunoblot of RCAN1 in atherosclerotic and non-atherosclerotic human coronary artery and in human internal mammary artery (hIMA). Protein bands corresponding to RCAN1-1 and RCAN1-4 are indicated. α-ACTIN was used as loading control.
2. The aortic sinus of *Apoe*^*−/−*^ mice fed standard chow or a high-fat diet (HFD) was immunostained for Rcan1. Representative images are shown of Rcan1 immunohistochemistry (top panels) and fluorescence microscopy (bottom panels). Bar, 50 µm.
3. Representative confocal immunofluorescence images of Rcan1 (red), lectin (green), αSMA (green) and Mac3 (green) in aortic sinus lesions of HFD-fed *Apoe*^*−/−*^ mice. Merged images are also shown. Bar, 5 µm.
4. Quantitative PCR analysis of *Rcan1* mRNA expression in the aortic arch of C57BL/6 and *Apoe*^*−/−*^ mice fed standard chow or an HFD. mRNA amounts were normalized to *Hprt1* expression (means ± SEM; *n* = 3). Student's *t*-test, *p = 0.013, **p = 0.0019.
5. Representative Rcan1 immunoblot in the same tissues as in D.

To investigate the role of RCAN1 in atherogenesis, we used the *Apoe*^*−/−*^ mouse model (Plump et al, 1992; Zhang et al, 1992). These mice develop atherosclerosis spontaneously, and the appearance of lesions is accelerated by feeding them a cholesterol-rich diet. Like human familial hypercholesterolemia patients, these mice develop lesions in the aortic valves (Getz & Reardon, 2012). We fed 3-month old *Apoe*^*−/−*^ mice a high-fat diet (HFD) for 6 weeks and compared Rcan1 expression in the aortic valves with that in wild-type and *Apoe*^*−/−*^ mice fed a standard chow diet. Aortic valves of *Apoe*^*−/−*^ mice fed an HFD showed marked Rcan1 staining in cells close to areas of lipid deposition, while staining was much weaker in the non-atherosclerotic valves of mice fed the control diet (Fig 1B, top panels). More intense Rcan1 expression in the valves of HFD-fed mice was also evident upon analysis by confocal immunofluorescence (Fig 1B, lower panels). Immunofluorescent staining of plaques for markers of endothelial cells, VSMCs and macrophages revealed elevated Rcan1 expression in all three cell types (Fig 1C). Quantitative PCR analysis of the aortic arch, which is also predisposed to lesion formation in mice, revealed higher *Rcan1* expression in chow-fed *Apoe*^*−/−*^ mice than in wt C57BL/6 mice, and expression was higher still in *Apoe*^*−/−*^ mice fed an HFD (Fig 1D). As in human samples, western blot analysis of Rcan1 expression in the aortic arch of these animals indicated that Rcan1-4 was strongly induced in atherosclerotic mice (Fig 1E). In contrast, Rcan1-1 expression was barely affected in atherosclerosis (Fig 1E).

**Figure 2:**
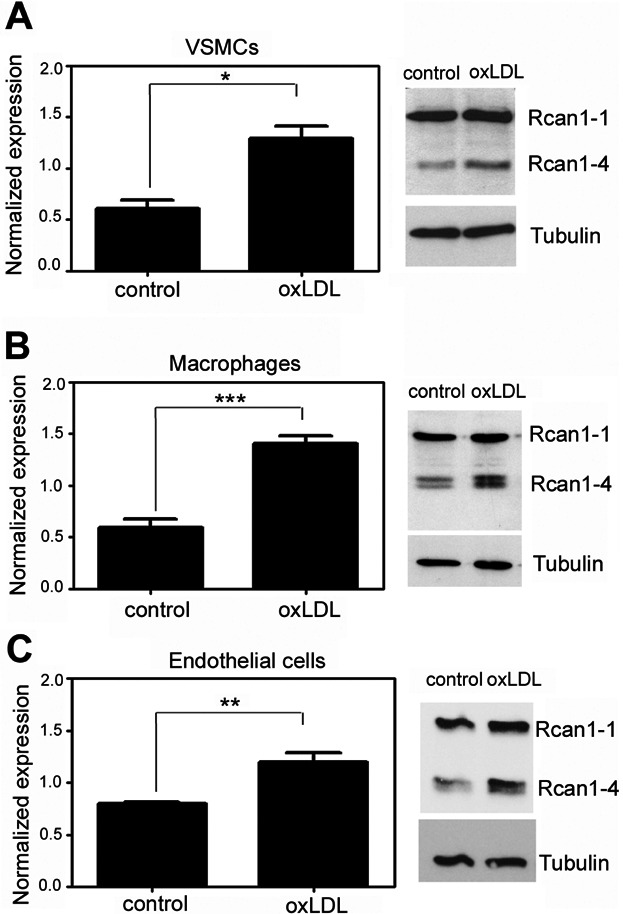
Induction of Rcan1-4 expression by oxLDL Quantitative PCR and representative immunoblot analysis of Rcan1 expression in cells isolated from *Apoe^−/−^* mice and treated with 50 µg/ml oxLDL:

The subendothelial accumulation of oxLDLs is a crucial early event in plaque formation. We therefore determined whether oxLDL regulates Rcan1 expression in the major cell compartments present in the plaque. Treatment of mouse primary macrophages, VSMCs, and endothelial cells with oxLDL induced the expression of *Rcan1* mRNA and Rcan1-4 protein in all three cells types, but did not induce Rcan1-1 protein (Fig 2).

1. VSMCs (*n* = 3),
2. Peritoneal macrophages (*n* = 3) and
3. Endothelial cells (*n* = 6). mRNA amounts were normalized to *Hprt1* expression (means ± SEM). Student's *t*-test, *p = 0.007, **p = 0.001, ***p = 0.0003.

### Rcan1 inactivation reduces atherosclerotic lesion burden

**Figure 3:**
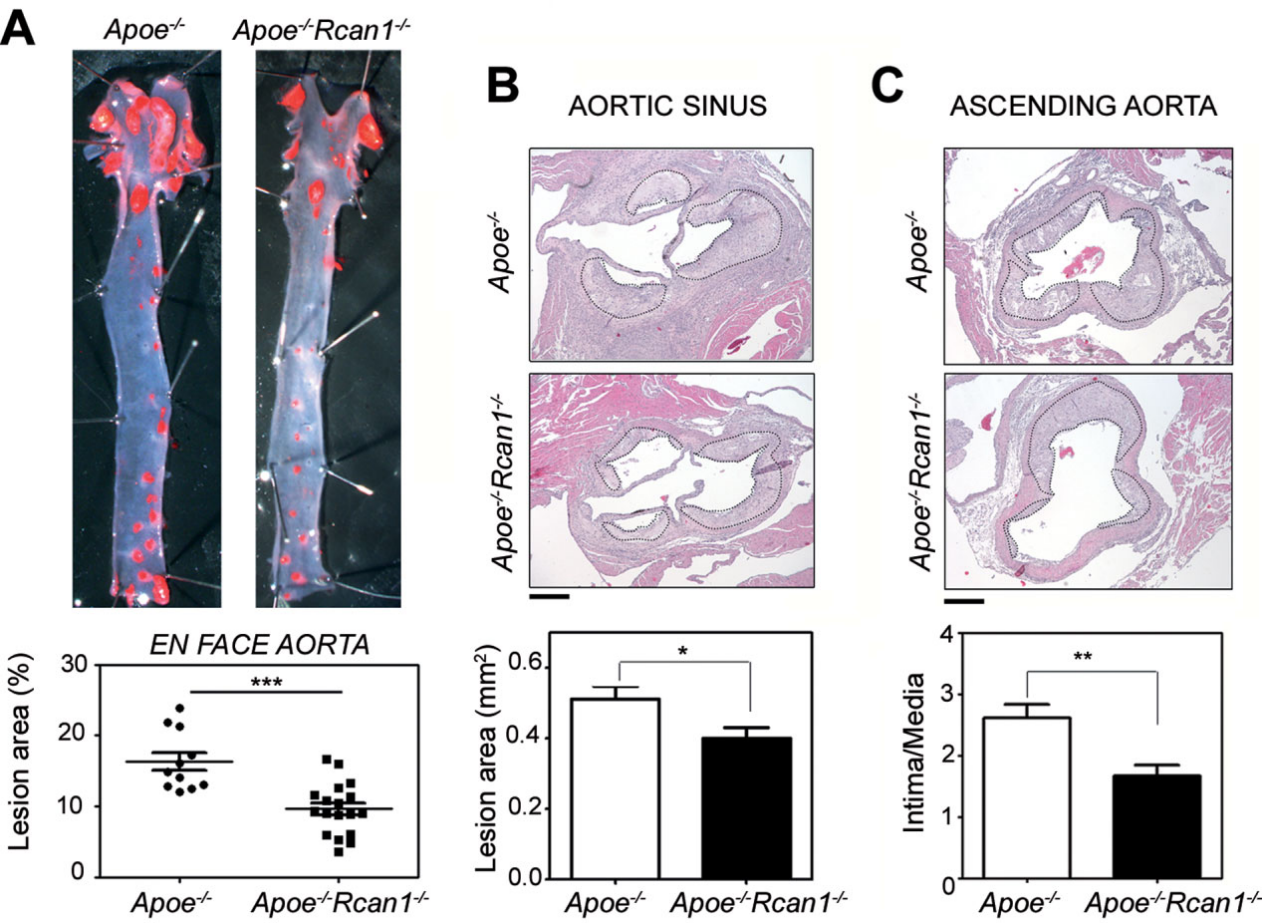
Rcan1 deficiency decreases atherogenesis burden

To investigate the contribution of Rcan1 to atheroma formation we compared *Apoe*^*−/−*^ with *Apoe*^*−/−*^*Rcan1*^*−/−*^ (double knockout) mice. *Rcan1* targeting affected both isoforms and lack of expression was confirmed in aorta and heart (Fig S2 of Supporting Information). *Apoe*^*−/−*^ and *Apoe*^*−/−*^*Rcan1*^*−/−*^ mice showed similar body weight increases after 6 weeks on an HFD (Fig S3A of Supporting Information), and there were no significant inter-group differences in serum concentrations of triglyceride, total and free cholesterol, high-density lipoprotein cholesterol and LDL cholesterol (Fig S3B–C of Supporting Information). *En face* analysis of Oil Red O stained atherosclerotic plaques revealed that aortic lesion size in *Apoe*^*−/−*^*Rcan1*^*−/−*^ mice was significantly smaller than in *Apoe*^*−/−*^ mice (Fig 3A). Since the aortic sinus and ascending aorta are particularly prone to atherosclerosis, we also compared atheroma formation in these regions. Haematoxylin and eosin (H&E) staining of cross-sections revealed a significantly smaller lesion area in both regions in *Apoe*^*−/−*^*Rcan1*^*−/−*^ mice (Fig 3B–C).

1. Representative Oil red O staining and quantification of lesion area in the aortas of *Apoe*^*−/−*^ (*n* = 11) and *Apoe*^*−/−*^*Rcan1*^*−/−*^ mice (*n* = 18) fed an HFD for 6 weeks. Each data point denotes an individual mouse, and the horizontal bars denote the mean (long bar) and the SEM.
2. Representative H&E staining of the aortic sinus and quantification of the lesion area in this region (mean ± SEM).
3. Representative H&E staining of the ascending aorta and quantification of lesion measured as intima/media ratio in this region in the same cohort of animals (mean ± SEM). Bars, 200 µm. Student's *t*-test, *p = 0.02, **p = 0.0025, ***p = 0.0001.

**Figure 4:**
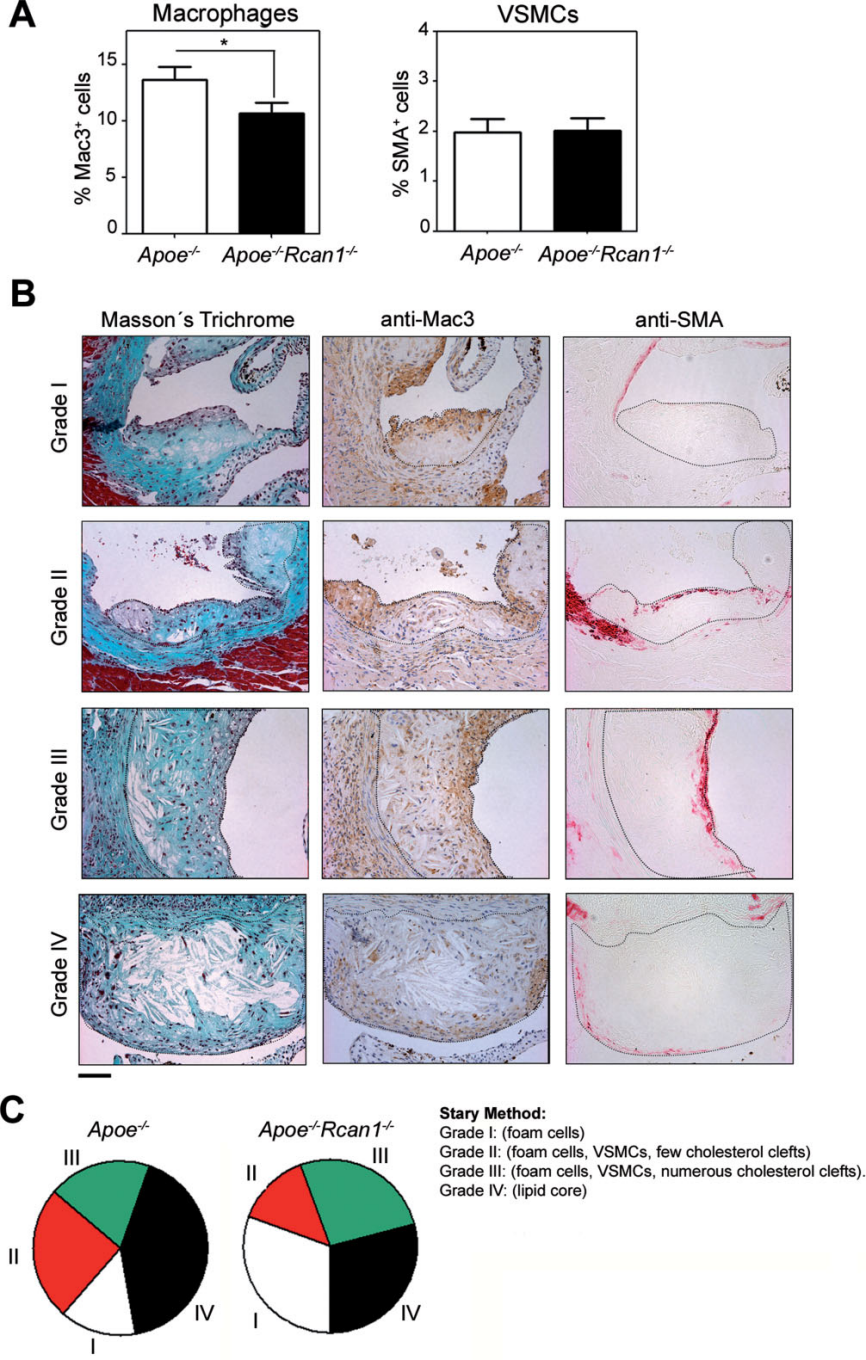
Rcan1 deficiency results in less-advanced plaques

To assess the progression of atheromas in *Apoe*^*−/−*^ and *Apoe*^*−/−*^*Rcan1*^*−/−*^ mice, we determined the presence of macrophages and VSMCs and the lipid deposition pattern in the aortic cusps, the region that contains the most advanced lesions in the *Apoe*-deficient model (Nakashima et al, 1994). The relative content of macrophages was lower in *Apoe*^*−/−*^*Rcan1*^*−/−*^ plaques, whereas numbers of VSMCs were similar in the two genotypes (Fig 4A). To confirm that *Apoe*^*−/−*^*Rcan1*^*−/−*^ mice had less-advanced plaques, we classified lesions according to the Stary method (Stary et al, 1994) into early plaques (grade I) containing only macrophages; grade II lesions containing macrophages, VSMCs and a few scattered cholesterol clefts; grade III lesions containing macrophages, VSMCs and numerous cholesterol clefts and advanced plaques (grade IV) containing macrophages, VSMCs and a large lipid core (Fig 4B). After 6 weeks of HFD, ≈ 42% plaques in *Apoe*^*−/−*^ mice were grade IV and only ≈ 14% were grade I (Fig 4C). In contrast, the proportion of grade IV plaques in *Apoe*^*−/−*^*Rcan1*^*−/−*^ mice was ≈ 28% and that of grade I plaques was ≈ 31% (Fig 4C). These results therefore indicate that Rcan1 plays a key role in atherosclerosis progression.

1. Quantification of neointimal macrophage and VSMC content in lesions of the aortic sinus of *Apoe*^*−/−*^ (*n* = 11) and *Apoe*^*−/−*^*Rcan1*^*−/−*^ mice (*n* = 18) fed an HFD for 6 weeks. Student's *t*-test, *p = 0.017.
2. Representative images of Masson's trichrome, anti-Mac3 and anti-SMA staining of lesions of the aortic sinus classified according to the Stary method: grade I (mostly foam cells), grade II (foam cells, VSMCs and few cholesterol clefts), grade III (foam cells, VSMCs and numerous cholesterol clefts) and grade IV (lipid core). Bars, 50 µm.
3. Classification of aortic sinus lesions according to the Stary method in the cohort of *Apoe*^*−/−*^ and *Apoe*^*−/−*^*Rcan1*^*−/−*^ animals shown in Fig 3.

**Figure 5:**
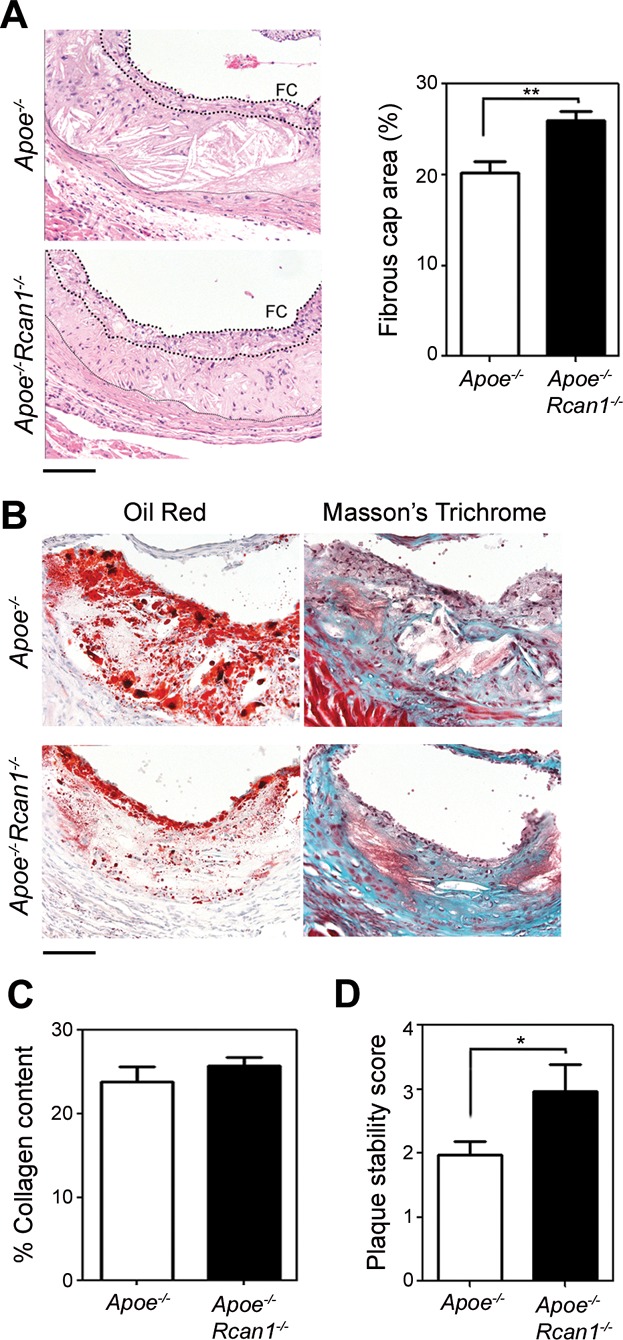
Rcan1 deficiency does not decrease plaque stability

While *Rcan1* targeting generates less advanced atherosclerotic plaques, it might as well potentially decrease their stability. Although the *Apoe*^*−/−*^ mouse is not particularly prone to develop unstable plaques in the aorta (Getz & Reardon, 2012), it was formally possible that Rcan1 deficiency might increase plaque vulnerability. We therefore determined the effect of Rcan1 targeting on plaque stability. A key feature of unstable plaques is thinning of the fibrous cap, usually accompanied by a reduction in collagen content. The extent and thickness of the fibrous cap, relative to plaque size, were larger in *Apoe*^*−/−*^*Rcan1*^*−/−*^ mice than in *Apoe*^*−/−*^ mice (Fig 5A and Fig S4 of Supporting Information), whereas the collagen content of the fibrous cap was similar in both genotypes (Fig 5B–C). Moreover, lipid content, determined by Oil Red staining, was lower in plaques of *Apoe*^*−/−*^*Rcan1*^*−/−*^ mice (Fig 5B) and these plaques had a higher stability score (Fig 5D). Expression of metalloproteases MMP2 and MMP9, another index of plaque instability, was almost identical in *Apoe*^*−/−*^ and *Apoe*^*−/−*^*Rcan1*^*−/−*^ plaques (Fig S5 of Supporting Information) and no plaques displayed evidence of haemorrhage (Fig S6 of Supporting Information). These data, together with the lower macrophage content of Rcan1-deficient plaques, thus indicate that *Rcan1* targeting does not induce characteristics of unstable atherosclerotic plaques, and suggest instead that Rcan1 inactivation might increase plaque stability.

1. Representative H&E staining and quantification of fibrous cap area of lesions in the aortic sinus of *Apoe*^*−/−*^ (*n* = 25 valves) and *Apoe*^*−/−*^*Rcan1*^*−/−*^ mice (*n* = 45 valves) fed an HFD for 6 weeks. Data are shown relative to the lesion area (means ± SEM). Student's *t*-test, **p = 0.0012. Thick and thin dotted lines, respectively, delimit the fibrous cap (FC) and lesion.
2. Representative images of Oil Red and Masson's trichrome staining of cryocut cross-sections of the aortic arch lesions of *Apoe*^*−/−*^ (*n* = 15) and *Apoe*^*−/−*^*Rcan1*^*−/−*^ mice (*n* = 17) fed an HFD for 6 weeks. Bar, 50 µm.
3. Quantification of collagen content in the fibrous cap of these lesions (mean ± SEM).
4. Plaque stability score determined by collagen content divided by Oil Red staining area of the same lesions (mean ± SEM; Student *t*-test, *p = 0.048).

### Rcan1 regulates CD36 expression and mediates foam-cell formation

**Figure 6:**
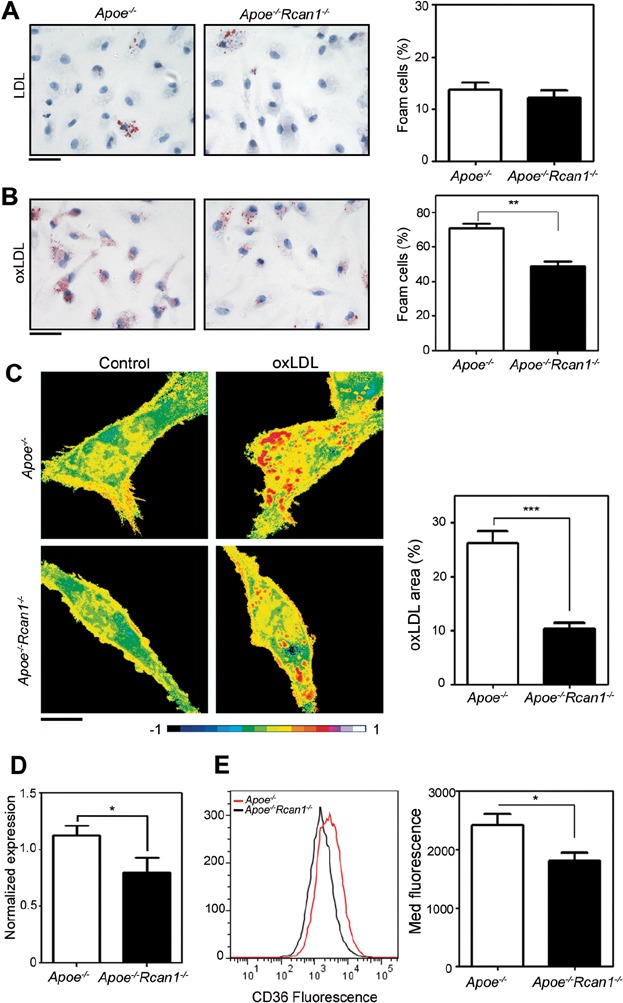
Rcan1 mediates macrophage uptake of oxLDL

Oil Red O staining of intracellular lipids indicated that while macrophages isolated from *Apoe*^*−/−*^ and *Apoe*^*−/−*^*Rcan1*^*−/−*^ mice engulfed unmodified LDL similarly (Fig 6A), fewer *Apoe*^*−/−*^*Rcan1*^*−/−*^ macrophages engulfed oxLDL, and *Apoe*^*−/−*^*Rcan1*^*−/−*^ macrophages also appeared to load less oxLDL per cell (Fig 6B). We measured intracellular oxLDL particles by Laurdan generalized polarization (Laurdan GP) (Sanchez et al, 2007; Sanchez et al, 2010), a confocal technique that measures water content inside lipid compartments. Intracellular oxLDLs are tightly packed and shift Laurdan GP towards red frequencies (Ferretti et al, 2002), whereas other lipid compartments, including intracellular membranes, contain more water and give a yellow or green signal (Sanchez et al, 2007; Sanchez et al, 2010). Macrophages exposed to oxLDL thus show its presence in the cytosol as red dots (Fig 6C). Quantification of the area occupied by oxLDL revealed that *Apoe*^*−/−*^ macrophages took up ≈ 2.5 times more oxLDL than *Apoe*^*−/−*^*Rcan1*^*−/−*^ cells (Fig 6C).

A,B. *Apoe*^*−/−*^ and *Apoe*^*−/−*^*Rcan1*^*−/−*^ peritoneal macrophages were exposed to (A) native LDL or (B) oxLDL for 24 h and then Oil Red O stained. Representative images and quantification are shown of stained cells in three independent experiments (mean ± SEM; Student's *t*-test, **p = 0.0018). Bars, 20 µm.
C. *Apoe*^*−/−*^ and *Apoe*^*−/−*^*Rcan1*^*−/−*^ peritoneal macrophages cultured in medium alone (control) or containing oxLDL were stained with Laurdan. Representative Laurdan GP images are shown with quantification of the area covered by oxLDL (red dots) (*n* = 20; mean ± SEM; Student's *t*-test, ***p = 0.0001). Black bar, 10 µm.
D. Quantitative PCR analysis of *CD36* expression in the aortic arch of atherosclerotic *Apoe*^*−/−*^ (*n* = 12) and *Apoe*^*−/−*^*Rcan1*^*−/−*^ mice (*n* = 13) pooled from three independent experiments. mRNA amounts were normalized to *m36B4* expression (means ± SEM). Student's *t*-test *p = 0.039.
E. Representative flow cytometry fluorescence histograms of CD36-stained macrophages of the indicated genotypes and quantification of the median fluorescence levels (*n* = 3; mean ± SEM; Student's *t*-test, *p = 0.036).

**Figure 7:**
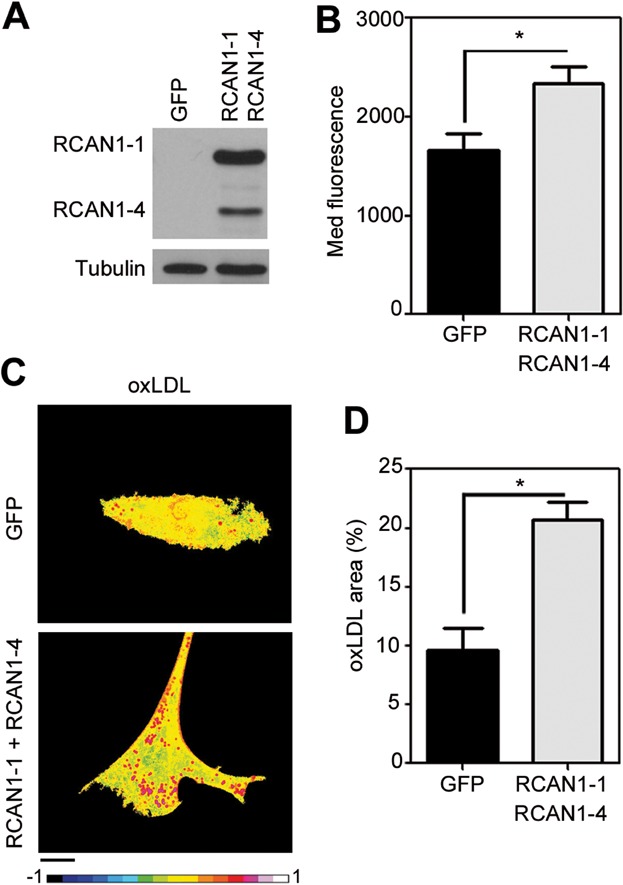
Rcan1 re-expression in *Apoe^−/−^Rcan1^−/−^* macrophages increases CD36 expression and the uptake of oxLDL *Apoe^−/−^Rcan1^−/−^* peritoneal macrophages were transduced with lentiviruses encoding GFP or Rcan1-1-IRES-GFP plus Rcan1-4-IRES-GFP.

OxLDL uptake and foam-cell formation are mediated by the class A and B scavenger receptors SR-A and CD36 (Febbraio et al, 2000; Suzuki et al, 1997). Real-time PCR analysis detected significantly higher levels of CD36 in the aortic arches of *Apoe*^*−/−*^ mice compared with *Apoe*^*−/−*^*Rcan1*^*−/−*^ mice (Fig 6D), whereas expression of SR-A was similar in the two genotypes (Fig S7A of Supporting Information). Accordingly, flow cytometry analysis revealed that CD36 levels, but not those of SR-A, were significantly downregulated in *Apoe*^*−/−*^*Rcan1*^*−/−*^ macrophages (Fig 6E and Fig S7B of Supporting Information). Lentiviral re-expression of Rcan1-1 and Rcan1-4 in *Apoe*^*−/−*^*Rcan1*^*−/−*^ peritoneal macrophages (Fig 7A) increased cell surface expression of CD36 (Fig 7B) and concomitantly increased the numbers of oxLDL particles taken up by *Apoe*^*−/−*^*Rcan1*^*−/−*^ peritoneal macrophages (Fig 7C–D). These results thus support that Rcan1 contributes to foam-cell formation by regulating CD36 expression.

A. Representative immunoblot analysis of Rcan1 expression in these cells.
B. Quantification of the normalized median fluorescence levels of CD36 determined by flow cytometry in the GFP^+^ population (*n* = 4; mean ± SEM; Student's *t*-test *p = 0.02).
C,D. (C) Representative Laurdan GP images and (D) quantification of the area covered by oxLDL (red dots) in GFP-F4/80 double-positive cells after culture in the presence of oxLDL (*n* = 3; mean ± SEM; Student's *t*-test, *p = 0.01). Scale bar, 10 µm.

Lipid accumulation by lesional macrophages can also reflect altered cholesterol efflux. To investigate the contribution of Rcan1 to cholesterol efflux, foam-cell formation was induced in *Apoe*^*−/−*^ and *Apoe*^*−/−*^*Rcan1*^*−/−*^ peritoneal macrophages by incubating them with particles of acetylated LDL (acLDL), a non-atherosclerotic modified form of LDL, in the presence of ^3^H-cholesterol. *Apoe*^*−/−*^*Rcan1*^*−/−*^ macrophages accumulated less ^3^H-cholesterol (Fig S8A of Supporting Information). Addition of HDL to the foam-cell cultures promoted cholesterol efflux, and this effect was modestly stronger in Rcan1-deficient cells (Fig S8B of Supporting Information). Consistent with this effect, ABC transporter expression was slightly higher in *Apoe*^*−/−*^*Rcan1*^*−/−*^ peritoneal macrophages and in atherosclerotic lesions in the aortic arch of these animals (Fig S8C–D of Supporting Information).

### Rcan1 regulates oxLDL-mediated inhibition of macrophage migration

**Figure 8:**
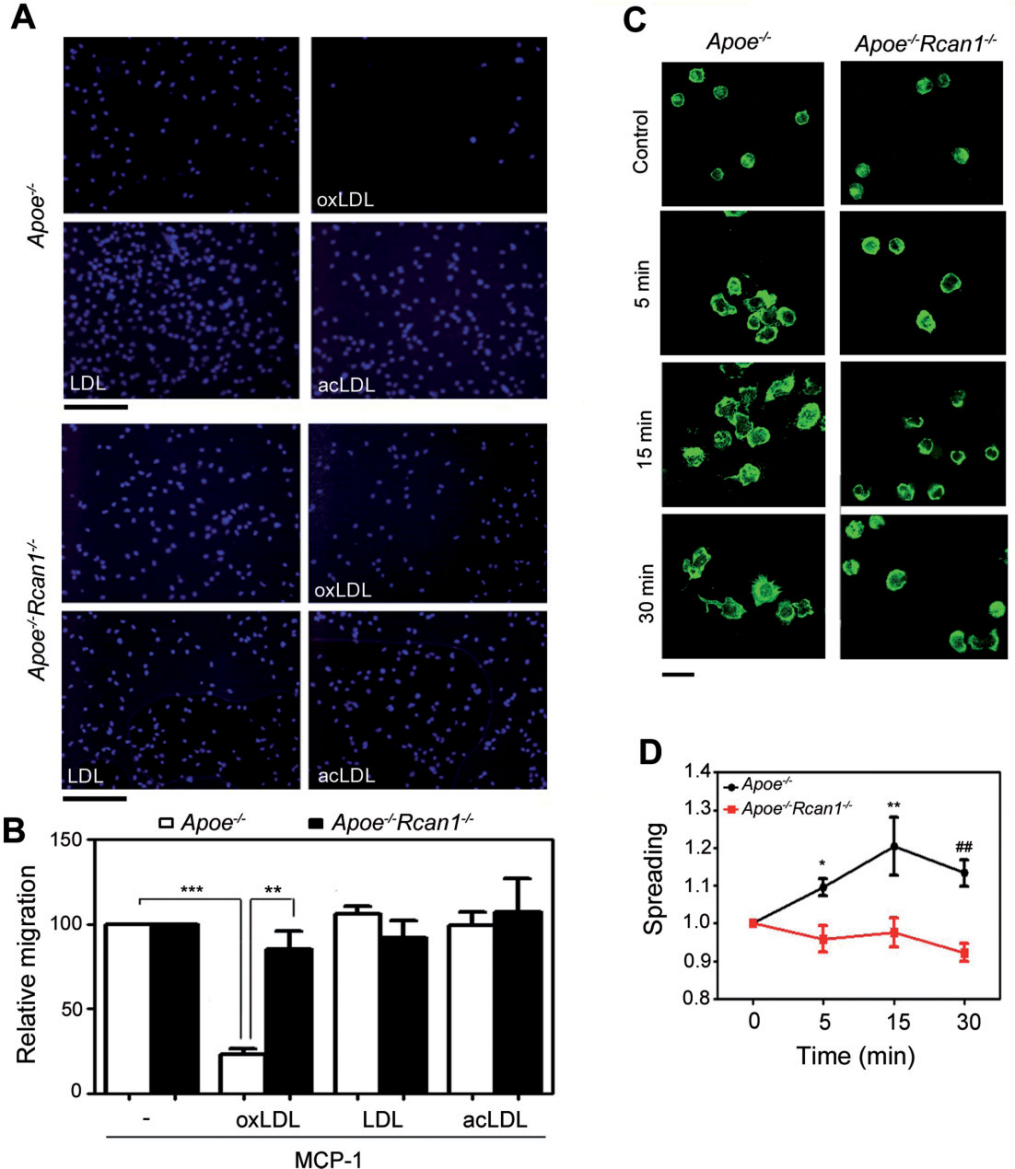
Macrophage migration is inhibited by oxLDL in an Rcan1-dependent manner

The presence of macrophages in atheroma plaques depends not only on their recruitment, but also on their capacity to exit the plaque, a process strongly impaired by oxLDL (Angeli et al, 2004; Randolph, 2008). Since Rcan1 can either promote or repress cell migration (Espinosa et al, 2009; Iizuka et al, 2004; Minami et al, 2004), we first determined whether Rcan1 was required for chemotactic macrophage migration. *Apoe*^*−/−*^ and *Apoe*^*−/−*^*Rcan1*^*−/−*^ macrophages migrated similarly in Boyden Transwell chambers in response to MCP-1 or a combination of MCP-1 and fetal bovine serum (FBS) (Fig S9 of Supporting Information). We next investigated whether Rcan1 participated in oxLDL-elicited inhibition of macrophage chemotaxis. While exposure of *Apoe*^*−/−*^ macrophages to oxLDL sharply reduced their migration towards the chemotactic stimulus, *Rcan1*-deficient macrophages were not affected (Fig 8A–B). Accordingly, in wound-healing assays oxLDL inhibited random migration of MCP1-treated *Apoe*^*−/−*^ macrophages, but not that of *Apoe*^*−/−*^*Rcan1*^*−/−*^ cells, as revealed by larger numbers of *Apoe*^*−/−*^*Rcan1*^*−/−*^ cells invading the denuded area (Fig S10 of Supporting Information). In the Transwell assays neither LDL nor acLDL significantly altered migration of macrophages of either genotype towards the chemoattractant (Fig 8A–B). These data thus suggest that Rcan1 does not directly regulate macrophage migration, but is a central mediator of oxLDL-elicited inhibition of their egress from atherosclerotic plaques.

A. Representative images of Hoechst-stained *Apoe*^*−/−*^ and *Apoe*^*−/−*^*Rcan1*^*−/−*^ macrophages, treated as indicated, on the lower filter surface of chemotaxis chambers (Bar, 100 µm).
B. Quantification of migrated macrophages in 10 fields per condition. Data are shown relative to non-treated cells (mean ± SEM, *n* = 4; one-way ANOVA, **p = 0.002, ***p < 0.00001).
C,D. *Apoe*^*−/−*^ and *Apoe*^*−/−*^*Rcan1*^*−/−*^ macrophages were plated on serum-coated coverslips, incubated with oxLDL at 37°C and then photographed at the indicated times. (C) Representative photomicroscopy images are shown (Bar, 20 µm) and (D) quantification of the spreading of these cells (mean ± SEM, *n* = 3; two-way ANOVA, *p = 0.019, **p = 0.002, ^##^*p* = 0.003).

Cell migration is a complex process that involves cellular spreading and cycles of formation and disruption of focal adhesion contacts (Stossel, 1994). To characterize the mechanisms by which Rcan1 mediates oxLDL-induced inhibition of macrophage chemotaxis, we measured its role in oxLDL-induced cell spreading. Spreading of *Apoe*^*−/−*^ macrophages, but not that of *Apoe*^*−/−*^*Rcan1*^*−/−*^ cells, was readily induced within 30 min of exposure to oxLDL (Fig 8C). Automated quantification of macrophage spreading revealed significant differences between *Apoe*^*−/−*^ and *Apoe*^*−/−*^*Rcan1*^*−/−*^ macrophages from early time points (Fig 8D).

### *Rcan1*^*−/−*^ macrophages express anti-inflammatory phenotype markers

**Figure 9:**
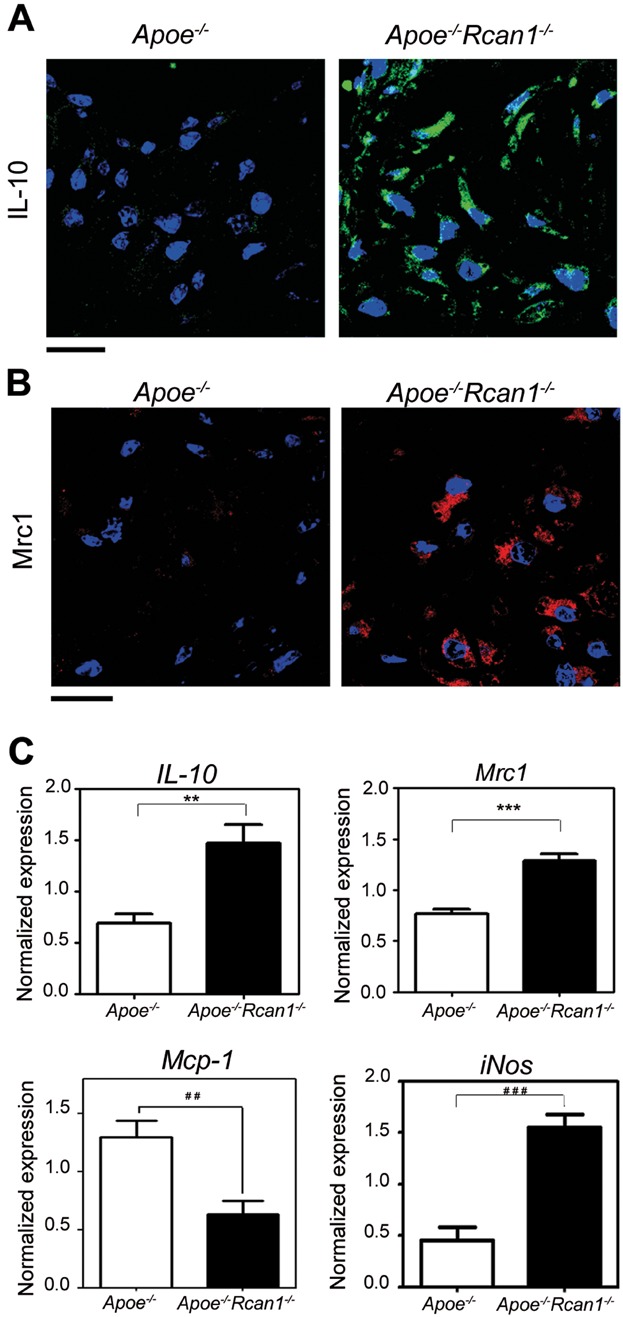
Increased expression of alternative macrophage polarization markers in *Apoe^−/−^Rcan1^−/−^* lesions and isolated macrophages

Although several types of macrophages have been detected in atherosclerotic lesions, macrophages with a classic M1-like pro-inflammatory phenotype appear to be the most abundant (Waldo et al, 2008). Given that oxLDL increases the expression of M1 markers (Chase et al, 2002) and *Apoe*^*−/−*^*Rcan1*^*−/−*^ macrophages ingest oxLDL less efficiently than *Apoe*^*−/−*^ macrophages, we postulated that atherosclerotic lesions of *Apoe*^*−/−*^*Rcan1*^*−/−*^ mice might contain macrophages with a phenotype different from those of *Apoe*^*−/−*^ mice. To test this, we analysed the expression of the anti-inflammatory phenotype markers Mrc1 and IL-10 in aortic cusps of *Apoe*^*−/−*^ and *Apoe*^*−/−*^*Rcan1*^*−/−*^ mice. Expression of both markers was higher in lesions from *Apoe*^*−/−*^*Rcan1*^*−/−*^ mice (Fig 9A–B), and *IL-10* levels were also higher in the aortic arch of atherosclerotic *Apoe*^*−/−*^*Rcan1*^*−/−*^ mice (Fig S11 of Supporting Information). These data suggest that lesions in *Apoe*^*−/−*^*Rcan1*^*−/−*^ mice are enriched in macrophages with a rather anti-inflammatory phenotype.

A,B. Representative immunofluorescence staining of (A) IL-10 and (B) Mrc1 in aortic sinus lesions of *Apoe*^*−/−*^ and *Apoe*^*−/−*^*Rcan1*^*−/−*^ mice fed an HFD. Bars, 20 µm.
C. Quantitative PCR analysis of *IL-10*, *Mrc1*, *Mcp-1* and *iNOS* mRNA expression in *Apoe*^*−/−*^ and *Apoe*^*−/−*^*Rcan1*^*−/−*^ peritoneal macrophages. mRNA amounts were normalized to *m36B4* expression (mean ± SEM; *n* = 3). Student's *t*-test, **p = 0.0078, ^##^*p* = 0.0058, ***p = 0.0007, ^###^*p* = 0.0009.

We next investigated the possible participation of Rcan1 in macrophage polarization. Expression of *IL-10* and *Mrc1* was significantly higher in *Apoe*^*−/−*^*Rcan1*^*−/−*^ macrophages compared with *Apoe*^*−/−*^ cells (Fig 9C), whereas *Apoe*^*−/−*^*Rcan1*^*−/−*^ macrophages expressed lower levels of *Mcp-1* levels (Fig 9C). Further consistent with an anti-inflammatory phenotype, *Apoe*^*−/−*^*Rcan1*^*−/−*^ macrophages showed higher expression of *Arg1* (Fig S12A of Supporting Information), modestly lower antigen presentation (Fig S12B of Supporting Information) and higher phagocytic activity (Fig S12C of Supporting Information). *Apoe*^*−/−*^*Rcan1*^*−/−*^ cells also expressed higher levels of *iNos*, a marker of pro-inflammatory M1 macrophages (Fig 9C), but *iNos* expression in atherosclerotic lesions of the aortic arch was almost identical in *Apoe*^*−/−*^ and *Apoe*^*−/−*^*Rcan1*^*−/−*^ mice (Fig S13 of Supporting Information). It thus seems that Rcan1 ablation might contribute to iNos induction by thioglycolate *ex vivo*, but not to its induction by proatherogenic stimuli *in vivo*.

### Transplantation of *Rcan1*^*−/−*^ BM cells confers resistance to atherosclerosis

To test the possible promotion of atherosclerosis by macrophage-expressed Rcan1, we reconstituted the BM of 2-month old lethally irradiated *Apoe*^*−/−*^ mice with BM-derived cells from *Apoe*^*−/−*^*Rcan1*^*−/−*^ or *Apoe*^*−/−*^ mice, thereby generating *Apoe*^*−/−*^ mice with either *Apoe*^*−/−*^*Rcan1*^*−/−*^ macrophages (*Rcan1*^*−/−*^ → *Apoe*^*−/−*^) or *Apoe*^*−/−*^*Rcan1*^*+/+*^ macrophages (*Rcan1*^*+/+*^ → *Apoe*^*−/−*^). Since RCAN1 has been reported to induce GSK3β expression (Ermak et al, 2006) and this protein modulates haematopoietic progenitor cell function (Lapid et al, 2013), it is formally possible that Rcan1 ablation influences the nature of BM reconstitution. However, chimeric *Rcan1*^*−/−*^ → *Apoe*^*−/−*^ mice showed no significant reduction in Gsk3β expression (Fig S14A of Supporting Information), and blood cell populations of reconstituted *Rcan1*^*+/+*^ → *Apoe*^*−/−*^ and *Rcan1*^*−/−*^ → *Apoe*^*−/−*^ mice were indistinguishable (Fig S14B–D of Supporting Information).

**Figure 10:**
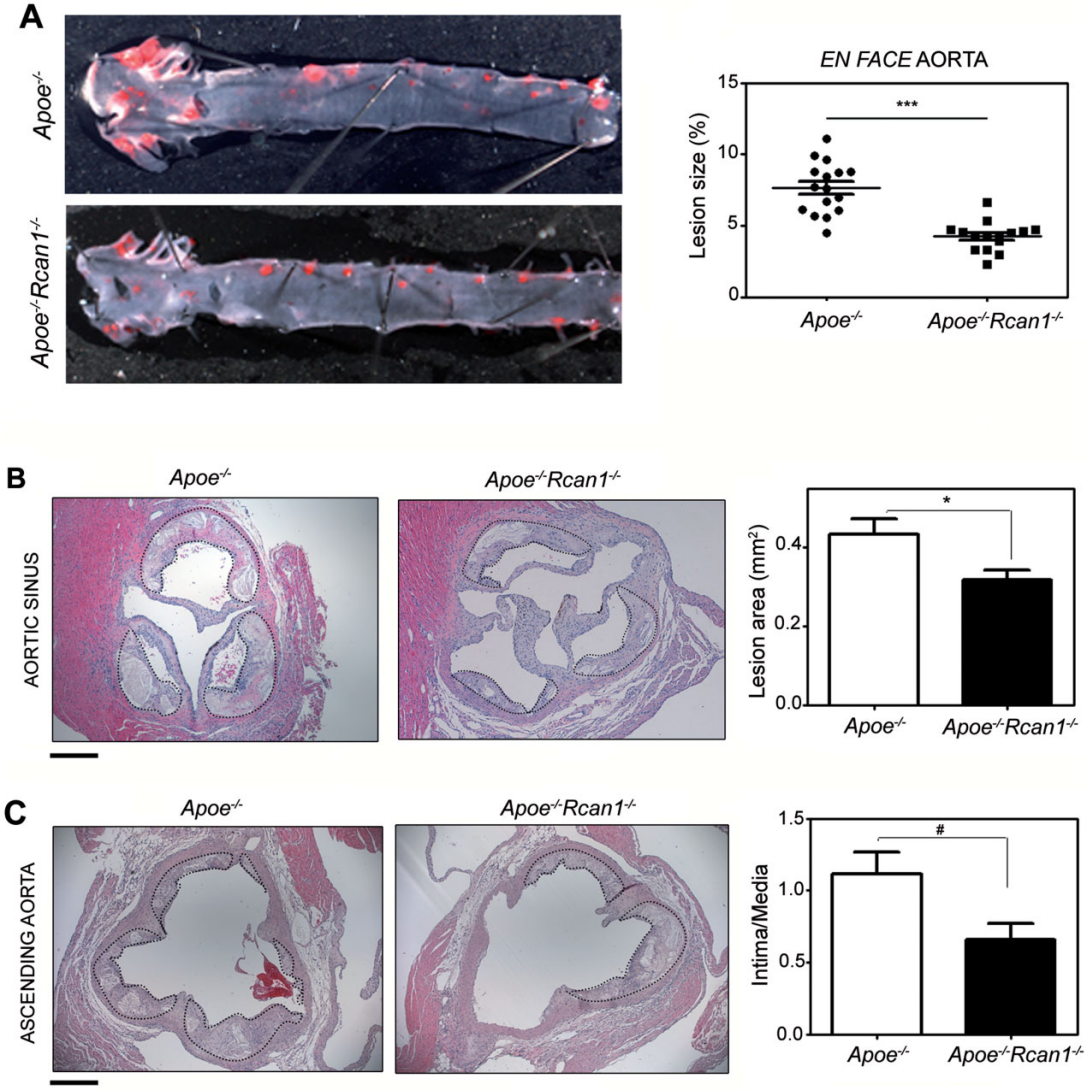
Atherosclerosis requires Rcan1 expression in BM-derived cells

After reconstitution for 4 week, mice were fed an HFD for 6 weeks. Chimeric *Rcan1*^*−/−*^ → *Apoe*^*−/−*^ and *Rcan1*^*+/+*^ → *Apoe*^*−/−*^ mice showed no significant differences in the body weight gain or in the serum concentrations of triglyceride, total cholesterol, HDL or LDL (Fig S15 of Supporting Information). *En face* analysis of Oil Red O aorta staining revealed markedly smaller lesion area in mice lacking expression of Rcan1 in BM-derived cells (Fig 10A). Cross-sectional analysis by H&E staining indicated that lesion size in the aortic sinus and the intima/media ratio in the ascending aorta were significantly smaller in *Rcan1*^*−/−*^ → *Apoe*^*−/−*^ mice than in *Rcan1*^*+/+*^ → *Apoe*^*−/−*^ mice (Fig 10B–C). These data demonstrate that *Rcan1* expression in the haematopoietic cell compartment plays a major role in atherosclerosis progression.

1. Representative Oil red O staining and quantification of the lesion area in the aortas of *Apoe*^*−/−*^ mice transplanted with BM from *Apoe*^*−/−*^ (*n* = 16) or *Apoe*^*−/−*^*Rcan1*^*−/−*^ (*n* = 15) mice after 6 weeks on an HFD. Each data point denotes an individual mouse, and the horizontal bars denote the mean (long bar) and the SEM.
2. Representative H&E images and lesion area quantification in aortic sinus sections of these mice. Quantification is shown as mean ± SEM (*n* = 13). Bar, 200 µm.
3. Representative H&E images and quantification of lesion measured as intima/media ratio. Data are means ± SEM (*n* = 12). Bar, 200 µm. Student's *t*-test, *p = 0.02, #*p* = 0.024, ***p = 0.0001.

## DISCUSSION

Atherosclerosis is a complex disease involving lipid accumulation and the central participation of endothelial cells, VSMCs and monocyte-derived macrophages. Our results demonstrate that RCAN1 is induced in human and mouse atherosclerosis and strongly suggest that Rcan1 promotes disease progression. In addition, we have identified several mechanisms underlying Rcan1-dependent atherosclerosis development. In particular, we have shown that Rcan1 mediates CD36 expression, foam-cell formation and oxLDL-elicited inhibition of macrophage migration. Moreover, *Rcan1* ablation promotes expression of IL-10 and other anti-inflammatory markers, including Mrc1, in macrophages and lesions.

Rcan1 plays a critical role in vascular wall remodelling associated with aneurysm and neointima formation after angioplasty; and notably, BM transplantation experiments showed that haematopoietic-cell expression of Rcan1 is not required for aneurysm (Esteban et al, 2011). In contrast, our current findings show that Rcan1 expression in BM-cells is critical for atherogenesis. Our results do not however exclude a proatherogenic role of Rcan1 in VSMCs or endothelial cells. The eventual production of tissue-specific *Rcan1*-targeted mice will help to resolve this question. The different requirements for BM-cell expression of Rcan1 in atherosclerosis and aneurysm might be attributable to the pivotal role of Rcan1 in foam-cell formation, a central feature of atherosclerosis but not of aneurysm.

Although human and mouse atherosclerosis differ in several aspects, critical features are shared, and our results indicate that one such feature is RCAN1 induction. Moreover, we demonstrate that oxLDL, one of main stimuli of atherosclerosis, strongly induces Rcan1-4 not only in macrophages and VSMCs, but also in endothelial cells. Although most leukocytes in human and mouse atherosclerotic lesions are macrophages, plaques also contain lymphocytes, neutrophils and mast cells (Woollard & Geissmann, 2010). Whether Rcan1 is also induced in these cells remains to be determined.

Whereas RCAN1-1 is usually constitutive, RCAN1-4 expression is induced by several stimuli. Our data suggest that both RCAN1 isoforms might be upregulated in atherosclerotic lesions. However, only Rcan1-4 appears to be upregulated by atherogenic oxLDL in macrophages, endothelial cells and VSMCs. The presence of RCAN1-1 in lesions might indicate that *in vivo* this isoform is induced together with RCAN1-4 in macrophages, endothelial cells or VSMCs. Alternatively, the high RCAN1-1 levels in lesions might reflect recruitment of other cell populations with constitutively high RCAN1-1 expression.

Our findings provide *in vivo* and *in vitro* evidence that Rcan1 is pro-atherogenic and that its genetic ablation deactivates several important pathological mechanisms. Rcan1 deficiency inhibits macrophage engulfment of oxLDL and hence foam-cell formation, and *Rcan1*^*−/−*^ macrophages are resistent to oxLDL-mediated inhibition of migration towards chemotactic stimuli, a feature related to foam-cell egress from the plaque *in vivo*. Finally, in the absence of Rcan1, macrophages appear to acquire features of a predominantly anti-inflammatory phenotype. Consistent with this view, *Rcan1* inactivation in the *Apoe*-deficient mouse atherosclerosis model attenuates atherosclerotic lesion burden in terms both of lesion area and severity. While most lesions of *Apoe*^*−/−*^ mice were advanced (grade IV), *Apoe*^*−/−*^*Rcan1*^*−/−*^ mice exhibited a greater number of early lesions (grade I) without showing any evidence of increased plaque instability.

The lower Oil Red O surface staining and the lower content of extracellular lipids in atherosclerotic lesions of *Apoe*^*−/−*^*Rcan1*^*−/−*^ mice are consistent with the relatively low uptake of oxLDL by *Apoe*^*−/−*^*Rcan1*^*−/−*^ macrophages. Interestingly, these macrophages have a similar capacity to engulf native LDL. Given that endocytic uptake of native LDL and oxLDL occurs via different receptors (Greaves & Gordon, 2009), our data suggest that Rcan1 promotes the expression or activity of receptors specific for chemically modified forms of LDL. This conclusion is supported by our findings that CD36, a scavenger receptor involved in the uptake of modified forms of LDL and foam-cell formation (Febbraio et al, 2000), is downregulated in *Rcan1* deficient macrophages, while re-expression of Rcan1-1 and Rcan1-4 in these cells increased CD36 expression and foam-cell formation. Cholesterol efflux was weakly increased in *Apoe*^*−/−*^*Rcan1*^*−/−*^ macrophages and the levels of the cellular transporters involved in this process were not significantly increased in these cells or in atherosclerotic lesions. Thus, the contribution of cholesterol efflux to Rcan1-mediated regulation of foam-cell formation remains uncertain.

The inflammatory nature of atherosclerotic disease is widely accepted. During the resolution of inflammation, macrophages egress from the inflamed site after engulfing pathogens, toxins or apoptotic cells. However, cholesterol-engorged macrophages fail to egress after clearing lipids and hence fail to resolve the inflammatory process (Angeli et al, 2004). Egress of macrophages from plaques is actively inhibited during hypercholesterolemia and this inhibition has been attributed, at least in part, to oxLDL loading into macrophages (Park et al, 2009). Our data suggest that Rcan1 might facilitate the trapping of lipid-laden macrophages in atherosclerotic lesions by mediating oxLDL uptake, macrophage spreading and inhibition of their egress. Our data thus suggest that Rcan1 might participate in a positive feedback loop in which Rcan1-mediated accumulation of oxLDL particles inhibits foam-cell egress from the plaque, resulting in increased exposure of the trapped foam cells to oxLDL and increased engulfment of these particles.

Most lesional macrophages in advanced plaques display a proinflammatory M1 phenotype, whereas alternatively activated M2 macrophages are more abundant in early lesions (Khallou-Laschet et al, 2010; Waldo et al, 2008). An additional macrophage subtype, whose gene expression program differs from that of M1 and M2 cells and which shows enhanced phagocytotic and chemotactic capacities, has also been reported in advanced plaques (Kadl et al, 2010), and analysis of CD163 and CD206 (MRC1) distribution in human carotid plaque macrophages also suggested that there may be at least three macrophage phenotypes present in human plaques (Bouhlel et al, 2007). Our results show elevated expression of IL-10 and Mrc1 in lesions of Rcan1 deficient mice and no increase of *iNos*. Since IL-10 is a powerful anti-inflammatory cytokine and its genetic ablation in mice promotes atherosclerotic lesion formation (Mallat et al, 1999), these results suggest that Rcan1 deficiency promotes the presence of macrophages with anti-inflammatory properties in the atheroma plaque, a conclusion consistent with the retarded progression of lesions in *Apoe*^*−/−*^*Rcan1*^*−/−*^ mice. Whether these IL-10-producing cells are M2-like macrophages or an additional macrophage type remains to be determined. Supporting an M2-like identity, *Apoe*^*−/−*^*Rcan1*^*−/−*^ macrophages expressed high levels of *Mrc1* and *arginase-1* and low levels of *Mcp1*, and showed elevated phagocytic activity and a low antigen presentation capacity. However, M2 macrophages appear to take up oxLDL more efficiently than M1 cells (van Tits et al, 2011), whereas *Apoe*^*−/−*^*Rcan1*^*−/−*^ macrophages engulfed less oxLDL than *Apoe*^*−/−*^ cells. Together, our data suggest that Rcan1 deficiency promotes a macrophage polarization distinct from M1 and M2.

The precise molecular mechanism involved in Rcan1 regulation of oxLDL uptake, foam-cell trapping and macrophage polarization has yet to be identified. Rcan1 was first identified as a negative regulator of CN activity (Rothermel et al, 2000). However, additional studies indicated that Rcan1 can also activate CN (Kingsbury & Cunningham, 2000; Vega et al, 2003) and our previous studies showed that Rcan1 neither activates nor inhibits CN in aortic tissues or primary VSMC cultures (Esteban et al, 2011). Our present results show that the absence of Rcan1 has no significant effect on CN activity in unstimulated cells or lipopolysaccharide-stimulated macrophages (Fig S16 of Supporting Information). Rcan1 thus appears to promote atherosclerosis without interfering with CN activity, perhaps through its interaction with other Rcan1-interacting proteins implicated in gene activation, such as Raf-1 (Cho et al, 2005), 14-3-3 (Abbasi et al, 2006) and NF-kB-inducing kinase (Lee et al, 2008).

**Figure 11:**
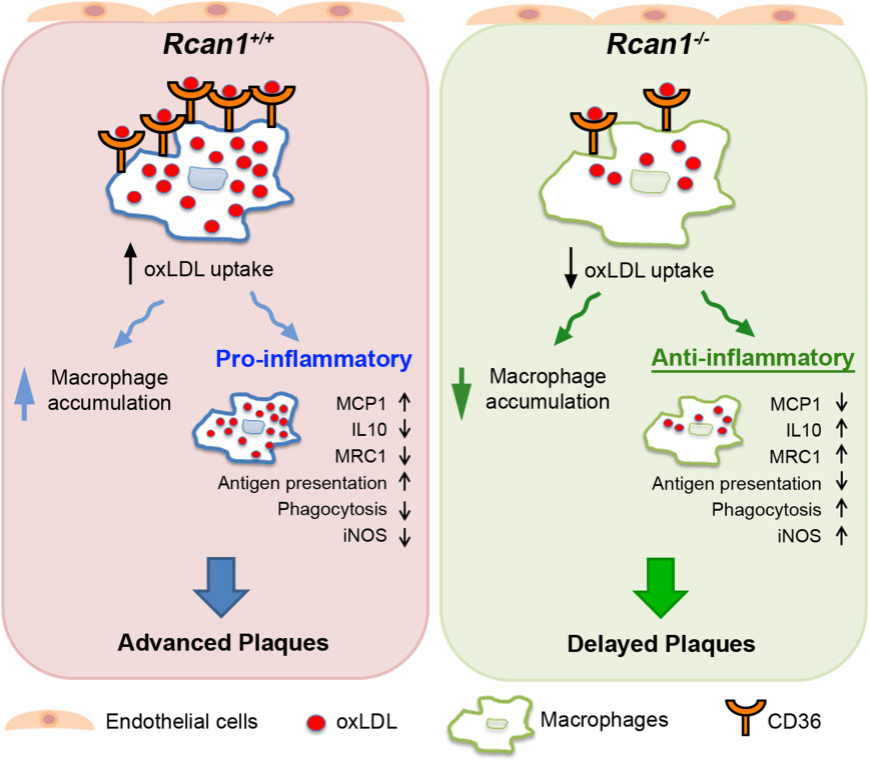
Model depicting the contribution of Rcan1 to plaque formation Rcan1 mediates expression of the oxLDL receptor CD36 in macrophages, thus regulating oxLDL uptake. Low uptake of oxLDL by Rcan1-deficient macrophages results in macrophage polarization to a phenotype distinct from M1 or M2. These cells display numerous anti-inflammatory features, including high levels of IL10 and MRC1, low levels of MCP1, pronounced phagocytosis activity and weak antigen presentation (Ag pres). However, these macrophages also show increased iNOS expression. Rcan1 ablation also decreases macrophage accumulation, likely by preventing their entrapment and/or the MCP1-mediated recruitment of additional macrophages. The lower accumulation of macrophages in the plaque and their conversion into predominantly anti-inflammatory cells might account for the delayed plaque progression.

Our results show that RCAN1 is induced in human and mouse atherosclerotic vessels, and suggest that Rcan1 regulates CD36 expression and thus contributes to the uptake of pathogenic forms of LDL cholesterol by macrophages in the plaque (Fig 11). The lower lipid uptake by Rcan1-deficient cells would attenuate their differentiation into pro-inflammatory macrophages and their accumulation in the plaque (Fig 11). Instead, expression by Rcan1-deficient macrophages of the anti-inflammatory cytokine IL-10 might inhibit further expansion of the plaque (Fig 11). These findings identify RCAN1 as an important regulator of atherosclerosis and strongly suggest that therapies aimed at inhibiting RCAN1 expression or function might significantly reduce atherosclerosis burden.

## MATERIALS AND METHODS

An expanded Materials and Methods Section is available in the Supporting Information.

### Animal procedures

Animal studies were in accordance with the guidelines of the EU on animal care and approved by the institutional ethics committee. Double-knockout *Apoe*^*−/−*^*Rcan1*^*−/−*^ mice were previously described (Esteban et al, 2011). *Rcan1* targeting constitutively ablates the expression of both *Rcan1-1* and *Rcan1-4* in every cell (Porta et al, 2007). To accelerate atherosclerosis, 3-month-old mice were fed an HFD (10.8% total fat, 0.75% cholesterol) for 6 weeks. BM transplantation was performed as previously described (Esteban et al, 2011). After 4 weeks on chow diet, transplanted mice were placed on the HFD for 6 weeks. Plasma concentrations of free cholesterol, total cholesterol, LDL-cholesterol, HDL-cholesterol and triglycerides were measured enzymatically.

### Human samples

Human coronary arteries and internal mammary arteries were collected from patients undergoing heart transplant and coronary artery bypass-graft surgery, respectively, at the Hospital de la Santa Creu i Sant Pau (Barcelona, Spain). The studies were approved by the Ethics Committee and were conducted in accordance with the Helsinki Declaration.

### Histological analysis

Cross-sections (5-µm) of paraffin-embedded or cryopreserved samples of mouse hearts and aortae were immunostained or evaluated by conventional Masson's Trichrome, H&E or Oil Red staining. Cross-sections were stained with antibodies and reagents specific for Rcan1, SMA, Mac3, Mrc1, IL-10, Ter-119 or endothelial cells and processed for either immunohistochemistry or immunofluorescence using standard procedures.

### Cell procedures

VSMCs were extracted from abdominal and thoracic aortas. Mouse lung endothelial cells were obtained from lung by selection with magnetic beads. Mouse peritoneal macrophages were collected by peritoneal lavage. Before stimulation, cells were rendered quiescent by culture in DMEM without FBS.

### Western blot analysis

Human specimens and mouse aorta samples for western blotting were snap-frozen in liquid nitrogen and stored at −80°C. Protein extracts were obtained by tissue lysis in ice-cold lysis buffer, separated under reducing conditions on SDS-polyacrylamide gels and transferred to nitrocellulose membranes. Proteins were detected with anti-Rcan1, anti-Gsk3-β, anti-α-actin, anti-alpha-tubulin, anti-PSF primary antibodies and HRP-conjugated secondary antibodies. Immunocomplexes were detected by chemiluminescence.

### Atherosclerotic lesion analysis

Hearts from euthanized mice were perfused through the left ventricle with PBS. After fixing in 4% paraformaldehyde overnight at 4°, the aortas were thoroughly cleaned to remove all adventitial fat and connective tissue. Aortas were whole-mount stained with 0.2% Oil Red O in 80% methanol, opened longitudinally and pinned to black wax to expose the entire luminal surface. Images were acquired and the area of atherosclerotic plaques was measured using ImageJ software.

### Migration assays

Migration of peritoneal macrophages was measured in a modified Boyden chamber using Transwell inserts with a 5 µm-pore membrane. Cells (1–2 × 10^5^ per well) in AlphaMEM supplemented with 0.1% BSA were loaded into the migration chamber with 50 µg/ml of lipoprotein (LDL, oxLDL or acLDL). The number of migrated cells was counted on fluorescence microscopy photographs of 10 randomly selected fields.

For wound healing assays, a single scrape wound was made on peritoneal macrophage monolayers. After washing with PBS, cells were incubated with 2% FBS plus 100 ng/ml MCP-1 with or without 50 µg/ml oxLDL. Macrophage motility was monitored by time-lapse videomicroscopy. The number of cells that migrated into the denuded area was counted using ImageJ.

### Spreading assays

Peritoneal macrophages were placed on serum-coated slides and allowed to attach. After stimulation with 50 µg/ml oxLDL, macrophages were fixed in 4% paraformaldheyde and stained with fluorescein-conjugated phalloidin. Cell perimeter and surface area were measured to determine cell spreading according to the formula


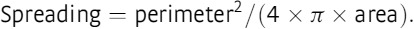


### Foam-cell formation

Peritoneal macrophages were plated on coverslips, incubated with 50 µg/ml LDL or oxLDL for 24 h, fixed in 4% paraformaldehyde and stained with Oil red O and counterstained with haematoxylin.

### Laurdan GP microscopy

Laurdan GP microscopy has been described previously (Bagatolli et al, 2003; Sanchez et al, 2007). Peritoneal macrophages were cultured in the presence of 1 µM Laurdan in serum-free medium. Laurdan fluorescence was excited with a mode-locked titanium-sapphire laser set at 780 nm and its emission collected at 445–465 nm and 474–514 nm. GP images were obtained with an ALBA microscope equipped with a 63× water objective and analysed using Image-J software.

### Quantitative PCR

Real-time quantitative RT-PCR was performed using a Prime Time qPCR assay specific for human *GAPDH* and TaqMan Gene Expression assays specific for human *RCAN1-1*, *RCAN1-4* and mouse *Rcan1* and *Hprt1*. SYBR Green was used for RT-PCR detection of mouse *CD-36*, *SR-A*, *ABCA*, *ABCG*, *IL-10*, *Mrc1*, *Arg1*, *Mcp-1*, *iNos* and *m36B4*. Calculations were made from measurements of 3 replicates of each sample. The amount of target mRNA in samples was estimated by the 2CT relative quantification method using *GAPDH*, *m36B4* or *Hprt1* for normalization.

### Statistical analysis

All values are expressed as means ± SEM. Differences were evaluated using one-way or two-way analysis of variance (ANOVA) and Bonferroni's *post hoc* test (experiments with ≥3 groups) or the Student's *t*-test, as appropriate for the data. Statistical significance was assigned at *p* < 0.05.

### The paper explained

PROBLEM:

Atherosclerosis, the underlying cause of myocardial infarction, stroke and peripheral vascular disease, is the major cause of morbidity and mortality in the developed world. It is a complex inflammatory disease characterized by accumulation of oxidized LDL (oxLDL) that triggers activation of the vascular endothelium and migration of monocytes into the lesion. These monocytes take up oxLDL and become lipid-laden foam cells that recruit smooth muscle cells and additional leukocytes. As RCAN1 is a signalling intermediate implicated in cell migration, we hypothesized that RCAN1 might contribute to atherosclerosis development.

RESULTS:

We show that RCAN1 is induced in atherosclerotic human vessels and in the atherosclerotic arteries of a mouse model of atherosclerosis. Rcan1 is expressed *in vivo* in lesional macrophages, endothelial cells and vascular smooth muscle cells and was induced *in vitro* by treatment of these cells with oxLDLs. *Rcan1* genetic deletion reduced the extent and severity of atherosclerosis in mice, and this effect was mechanistically linked to diminished expression of the oxLDL receptor in macrophages (CD36), decreased oxLDL uptake, resistance to oxLDL-mediated inhibition of macrophage migration and a shift of macrophage polarization towards an anti-inflammatory phenotype. Importantly, transplantation of Rcan1-deficient bone-marrow-derived cells greatly inhibited atherosclerosis.

IMPACT:Our data define a major role for haematopoietic Rcan1 in atherosclerosis and suggest that Rcan1 might facilitate the trapping of lipid-laden macrophages in atherosclerotic lesions by upregulating CD36-mediated oxLDL uptake and thereby trapping proinflammatory macrophages in atherosclerotic lesions. Our findings strongly suggest that future therapies aimed at inhibiting RCAN1 expression or function might significantly reduce atherosclerosis burden.

## Author contributions

The study was conceived by JMR and MRC. NMB, VE, JMR and MRC designed the study and analysed the data. NMB and VE performed most of the experiments with contributions from SV, AE, KU and AA. CR and JMG assessed RCAN1 expression in human samples. SAS performed Laurdan GP microscopy and analysed the data obtained. VA, TO and TM provided experimental support and ideas for the project. JMR and MRC wrote the manuscript with contributions of NMB, SAS and JMG. All authors read and approved the manuscript.

## Notes

As a service to our authors and readers, this journal provides supporting information supplied by the authors. Such materials are peer reviewed and may be re-organized for online delivery, but are not copy-edited or typeset. Technical support issues arising from supporting information (other than missing files) should be addressed to the authors.
