# Supplementary material for: A major role for RCAN1 in atherosclerosis progression
Source: EMBO Mol Med. 2013 Oct 15;5(12):1901–17. doi: 10.1002/emmm.201302842 (PMC3914525; doi:10.1002/emmm.201302842)
Supplement: Supplementary file 1 [file emmm0005-1901-sd1.pdf]

EMBO
Molecular Medicine

A major role for RCAN1 in atherosclerosis progression

Nerea Méndez-Barbero; Vanesa Esteban; Silvia Villahoz; Amelia Escolano; Katia Urso; Arantzazu Alfranca; Cristina Rodríguez; Susana A. Sánchez; Tsuyoshi Osawa; Vicente Andrés; José Martínez-González; Takashi Minami; Juan Miguel Redondo, and Miguel R. Campanero.

Corresponding author: Miguel Campanero, Instituto de Investigaciones Biomedicas

Review timeline:

Submission date:	04 April 2013
Editorial Decision:	08 May 2013
Revision received:	08 August 2013
Accepted:	03 September 2013

Transaction Report:

(Note: With the exception of the correction of typographical or spelling errors that could be a source of ambiguity, letters and reports are not edited. The original formatting of letters and referee reports may not be reflected in this compilation.)

Editor: Roberto Buccione

1st Editorial Decision 08 May 2013

Thank you for the submission of your manuscript to EMBO Molecular Medicine.

We experienced unusual difficulties in securing three appropriate reviewers in a timely manner. Since we cannot justify a further delay, I am making a decision based on the consistent evaluations of two Reviewers at this time.

You will see that while the Reviewers are generally supportive of you work, they express a number of concerns that prevent us from considering publication at this time. I will not dwell into much detail, as the evaluations are detailed and self-explanatory. I would like, however, to highlight a few main points.

Reviewer 1 is concerned that the data suggesting that RCAN1 primes macrophages towards a pro-inflammatory phenotype is not fully convincing. S/he also feels that the model system is not sufficiently described and that further experimentation on isoform expression is necessary. Reviewer 1 also lists other issues that require your intervention and mentions a few items that should be better discussed or supported with improved statistical treatment.

Reviewer 2 appreciates the bone marrow transplantation studies but feels that further clarification as to how and if RCAN1 modifies the BM reconstitution of recipients. S/he also suggests transient RCAN1 knock-down and over-expression experiments in macrophages would afford additional insight into the link between CD36 and RCAN1. Finally, Reviewer 2 mentions that the translational value of your work would be improved by additional plaque characterisation, as indicated.

I would also like to take the opportunity to mention that as per our Author Guidelines, the description of all reported data that includes statistical testing must state the name of the statistical

test used to generate error bars and P values, the number (n) of independent experiments underlying each data point (not replicate measures of one sample), and the actual P value for each test (not merely 'significant' or 'P < 0.05').

Considered all the above, while publication of the paper cannot be considered at this stage, we would be prepared to consider a revised submission, with the understanding that the Reviewers' concerns must be fully addressed, with additional experimental data where appropriate and that acceptance of the manuscript will entail a second round of review.

Please note that it is EMBO Molecular Medicine policy to allow a single round of revision only and that, therefore, acceptance or rejection of the manuscript will depend on the completeness of your responses included in the next, final version of the manuscript.

As you know, EMBO Molecular Medicine has a "scooping protection" policy, whereby similar findings that are published by others during review or revision are not a criterion for rejection. However, I do ask you to get in touch with us after three months if you have not completed your revision, to update us on the status. Please also contact us as soon as possible if similar work is published elsewhere.

I look forward to recieving your revised version of this mansucript in the near future

***** Reviewer's comments *****

Referee #1 (Comments on Novelty/Model System):

Technical Quality- The experiments are performed to a high standard and the in vivo work is robust. a particular strength is the use of Laurdan GP micropscopy for analysis of foam cell formation.
Novely- Althoug the role of RCAN-1 in atherosclerosis is interesting it not evident if it plays a causal role as the mechanistic underpinnings of its regualtion in the context atherosclerosis have not been described.
Medical Impact- The data increases our understanding of mediators in the development of atherosclerosis. Whilst the data suggests that RCAN1 may prime macrophages towards a pro-inflammatory phenotype this is not convincing, specifically the increased iNOS expression in RCAN1 deficient is conflicting with the data showing increased IL-10.
Model systesm- Whilst the strategy for investigating the effect of RCAN1 on atherosclerosis is robust, the model is not well described and further characterisation of the expression of the isoforms in the model system need to be included in the manuscript.

Referee #1 (Remarks):

This manuscript describes a potential role for RCAN1 in the pathogenesis of atherosclerosis in vivo. The data presented suggests that this is in part due to altered macrophage function and alteration in macrophage phenotype. In general the manuscript is well written and the experimental analysis is performed to a high standard. Although the data are for the most part intriguing there are additional experiments required to show a direct role of RCAN1 on macrophage function, foam cell formation and on the regulation of macrophage scavenger receptors. In addition, the mechanism through which RCAN1 deletion inhibits progression of atherosclerosis is not defined.

1. The introduction lacks some important information regarding RCAN1 isoforms. Page 3-4: What is the homology between RCAN-1 and RCAN-4 in humans and mice? Furthermore, what exactly are the differential roles of RCAN1-1 and RCAN1-4 in pathological processes specifically those involved in the pathogenesis of cardiovascular and vascular disease. For example, although the authors state that RACN1 plays a role in neointimal formation, it is not clear to which isoform the authors refer. This is important in light of the data presented in Fig. 1 and Fig. 2 of the manuscript. Why are the opposing roles for RCAN-1 on cell migration due only to different cell type? Is it possible that this may be due to RCAN1 regulation or differential cellular expression of both isoforms. These needs to be clarified.
2. An accurate and detailed description of the mouse model employed in the experiments is missing.

Is the RCAN1-/- mouse deficient in one or both isoforms of RCAN1? The authors should include tissue expression of RCAN1-1 and RCAN1-4 in the aorta or heart of Apoe-/- and ApoE/RACN1 DKO animals.
Similarly, if the RACN-1 mouse is deficient in only one isoform, this needs to be explained and isoform expression shown.
3. Fig.1 . The findings using Western Blot analysis of RCAN1 protein expression in human atherosclerosis need to be validated by performing RT-PCR of both isoforms in human samples.
4. Fig. 5. Additional experiments need to be performed to show gene expression of CD36. What was the rationale for only investigating CD36? Flow cytometry analysis and gene expression of other scavenger receptors e.g. SRA-1 need to be included. Furthermore, although the data suggest that deletion of RCAN-1 inhibits oxLDL uptake the effect of cholesterol efflux should also be examined in macrophages from ApoE-/- and ApoE/RACN1 DKO animals. This is important. If the authors are correct and RCAN-1 mediates its effect via regulation of CD36 this needs to be further validated. Therefore, additional experiments showing the effect of overexpression of RCAN-1 on both foam cell formation and scavenger receptor expression need to be performed either in bone marrow derived (or peritoneal macrophages) or indeed in a relevant macrophage cell line.
5. The data presented in Fig 6A and 6B should be presented as a single graph so that direct comparisons between Apoe-/- and ApoE/RACN-1 DKO can be illustrated. In Fig. 6C is the assumption that the inhibition of macrophage spreading in ApoE/RACN-1 macrophages as a results of CD36 inhibition? Again, this needs to be validated in vitro where macrophage spreading in macrophages transfected with RCAN-1.
6. Additional data showing the differences in serum/plasma of Il-10 levels in the animals groups would support the IL-10 expression.
7. Discussion. The authors state that RCAN1 is induced in mouse atherosclerosis and promotes disease progression. The data presented does not directly show that increased RCAN-1 increases disease progression. Therefore, the aforementioned in vitro validation experiments will facilitate the authors to conclude that RCAN-1 alters macrophage function towards a pro-inflammatory phenotype.
8. The data suggests that RCAN1 may inhibit macrophage egress. Evidence for this in vivo would provide a significant contribution to the manuscript although in the absence of relevant models this may be difficult to achieve.
9. Supplementary Material. There is some degree of repetition between the supplementary methods and those in the main body of the manuscript for example the section on Laurdan GP microscopy (and indeed others) are reptitive and should be edited accordingly.
10. Supplementary Fig 1. Is there a significant decrease in body weight between ApoE-/- and ApoE/RACN1-/- before HFD? Perhaps this data would be better represented by showing % weight gain in the two animals groups.
11. Supplementary Figure 3. The authors indicate that macrophage motility is inhibited by oxLDL in a RCAN1 dependent manner. There are no statistical analysis to support this. Please include the p value for comparison between MCP-1 plus oxLDL in ApoE-/- and ApoE/RACN1-/-. Without this the data does not support the conclusion.
12. Supplementary Figure 4. Is the difference in Arg1 expression statistically significant? This is referred to in the main body of the text and should be validated and p values included.

Referee #2 (Remarks):

MÈndez-Barbero et al report that RCAN1 promotes atherogenesis via polarization toward a pro-inflammatory macrophage phenotype, possibly due to the induction of the oxidized-LDL scavenger receptor CD36. After observing increased RCAN1 expression in multiple cellular lineages within human atherosclerotic plaque, and corroborating this observation in ApoE-/- mice, they have defined the role of RCAN1 in atherogenesis using Rcan1 knockout mice crossed with ApoE-/-. Rcan1 loss is associated with a reduction in atheroma volume and complexity, along with reduced macrophage content. RCAN1 promoted oxidized-LDL uptake and mediated the inhibitory effect of oxidized-LDL upon macrophage migration in vitro. The authors suggest that the associated reduction in CD36 expression noted in Rcan1-/- macrophages explains many of their observations, although note that they cannot offer a mechanistic link between Rcan1 expression and CD36 expression. The importance of bone marrow derived cells to their observations is supported in a series of bone marrow transplantation studies with ApoE-/- recipients receiving donor material from

ApoE-/- (control) or Rcan1-/-ApoE-/- mice.

The authors' work is interesting, appears well conducted and the manuscript is clearly written. Suggestions to improve the manuscript include:

1) Until tissue specific Rcan1-/- mice are available, the presented bone marrow transplantation studies are important to more strongly implicate monocyte/macrophage RCAN1. It is therefore important that the authors clarify whether Rcan1 alters the bone marrow reconstitution of recipients, since this may also alter their findings. For example, RCAN1 has been suggested to stimulate GSK3b expression in some tissues (FEBS J. 2006 May;273(10):2100-9); GSK3b is also important in modulating hematopoietic stem/progenitor cell function (e.g. J Clin Invest. 2013 Apr 1;123(4):1705-17) so may influence the nature of bone marrow reconstitution.

2) Whilst the authors do not offer a mechanistic link between RCAN1 and CD36 expression, short term knockdown or over-expression of Rcan1 in macrophages may provide further support of a temporal relationship between altered expression of Rcan1 and CD36. Such studies may also allow further probing of the mechanistic link with CD36.

3) Since Rcan1-/- mice exhibit reduced vascular restenosis due to reduced vascular smooth muscle cell migration, one translational concern of RCAN1 targeting may be the generation of unstable atherosclerotic plaques. The manuscript would be improved if additional plaque characterization were included; indices such as collagen content, MMP expression, fibrous cap thickness and plaque hemorrhage may be useful.

1st Revision - authors' response 08 August 2013

Referee #1 (Comments on Novelty/Model System):

Technical Quality- The experiments are performed to a high standard and the in vivo work is robust. a particular strength is the use of Laurdan GP micropscopy for analysis of foam cell formation.
Novely- Althoug the role of RCAN-1 in atherosclerosis is interesting it not evident if it plays a causal role as the mechanistic underpinnings of its regualtion in the context atherosclerosis have not been described.
Medical Impact- The data increases our understanding of mediators in the development of atherosclerosis. Whilst the data suggests that RCAN1 may prime macrophages towards a pro-inflammatory phenotype this is not convincing, specifically the increased iNOS expression in RCAN1 deficient is conflicting with the data showing increased IL-10.
Model systesm- Whilst the strategy for investigating the effect of RCAN1 on atherosclerosis is robust, the model is not well described and further characterisation of the expression of the isoforms in the model system need to be included in the manuscript.

Referee #1 (Remarks):

This manuscript describes a potential role for RCAN1 in the pathogenesis of atherosclerosis in vivo. The data presented suggests that this is in part due to altered macrophage function and alteration in macrophage phenotype. In general the manuscript is well written and the experimental analysis is performed to a high standard. Although the data are for the most part intriguing there are additional experiments required to show a direct role of RCAN1 on macrophage function, foam cell formation and on the regulation of macrophage scavenger receptors. In addition, the mechanism through which RCAN1 deletion inhibits progression of atherosclerosis is not defined.

1. The introduction lacks some important information regarding RCAN1 isoforms. Page 3-4: What is the homology between RCAN-1 and RCAN-4 in humans and mice? Furthermore, what exactly are the differential roles of RCAN1-1 and RCAN1-4 in pathological processes specifically those involved in the pathogenesis of cardiovascular and vascular disease. For example, although the authors state that RACN1 plays a role in neointimal formation, it is not clear to which isoform the authors refer. This is important in light of the data presented in Fig. 1 and Fig. 2 of the manuscript. Why are the opposing roles for RCAN-1 on cell migration due only to different cell type? Is it

possible that this may be due to RCAN1 regulation or differential cellular expression of both isoforms. These needs to be clarified.

We thank the Reviewer for careful reading of our manuscript and raising these important points. We indicate in the revised version of the manuscript that human and mouse RCAN1-1 and RCAN1-4 are highly conserved (end of page 3 – beginning of page 4) and the revised Introduction text provides extended information on RCAN1 (page 4, first paragraph).

The role of Rcan1 in neointimal formation cannot be attributed to a single isoform because both isoforms were knocked-out in the mouse model used in those studies. Indeed, to the best of our knowledge, as yet there has been no report of specific gene targeting for only one Rcan1 isoform. Moreover, no reports on the effect of Rcan1 forced expression or knock-down on any activity have systematically addressed the effect of each isoform individually. As a result, it has not yet been possible to ascribe any of the Rcan1-mediated activities to a particular isoform. We now state in the manuscript "*Since most studies have involved the simultaneous inactivation of Rcan1-1 and Rcan1-4 or have examined the effect of over-expressing or knocking-down only one of these isoforms, it has not yet been possible to ascribe specific roles to one Rcan1 isoform and not the other.*" (page 4, end of first paragraph).

The *Rcan1*$^{-/-}$ mouse used in our studies was generated by deleting *Rcan1* exon 6 (Porta et al, 2007). This exon is common to both isoforms and its targeting ablates the expression of both (Porta et al, 2007). We previously showed that Rcan1 expression was required for migration of vascular smooth muscle cells (Esteban et al, 2011). Since these VSMCs lacked both isoforms, we could not formally attribute a specific role in cell migration to either of them. Although we also showed that the ectopic expression of Rcan1-4 in *Rcan1*$^{-/-}$ VSMCs rescued migration of these cells, we cannot attribute a pro-migratory role only to this isoform because we did not attempt to rescue the migratory phenotype with Rcan1-1. We concur with the reviewer that the opposing roles of Rcan1 on cell migration are not necessarily due only to cell type differences, and could also be attributed to differences in Rcan1 regulation or differential expression of its isoforms. We have therefore rewritten the corresponding sentence: "*Rcan1 thus appears to have opposite roles in cell migration in different settings*." (page 4, en of first paragraph).

2. An accurate and detailed description of the mouse model employed in the experiments is missing. Is the RCAN1-/- mouse deficient in one or both isoforms of RCAN1? The authors should include tissue expression of RCAN1-1 and RCAN1-4 in the aorta or heart of Apoe-/- and ApoE/RACN1 DKO animals.
Similarly, if the RACN-1 mouse is deficient in only one isoform, this needs to be explained and isoform expression shown.

We thank the reviewer for raising this issue. Since the *Apoe*$^{-/-}$*Rcan1*$^{-/-}$ mouse had been previously described, we only provided the corresponding reference. We now realize that this information would be of great help for readers. We have therefore indicated under Materials & Methods "*Rcan1 targeting constitutively ablates the expression of both Rcan1-1 and Rcan1-4 in every cell (Porta et al, 2007).*" (page 15, Animal procedures). In addition, the detailed Materials and Methods (Supporting Information) states "*Rcan1*$^{-/-}$ *mice (mixed C57BL/6 and 129P2/OlaHsd background) (Porta et al, 2007), in which both Rcan1-1 and Rcan1-4 are targeted simultaneously, were backcrossed through >6 generations on a C57BL/6J background (Esteban et al, 2011) and subsequently crossed with Apoe*$^{-/-}$ *mice to create double-knockout Apoe*$^{-/-}$*Rcan*$^{-/-}$ *mice (Esteban et al, 2011).*" (page 1 of Supporting Information).

In addition, following this reviewer's suggestion, we have included a new immunoblot showing expression of Rcan1-1 and Rcan1-4 in aortic and heart tissue of *Apoe*$^{-/-}$ mice and their complete absence in the same tissues of *Apoe*$^{-/-}$*Rcan1*$^{-/-}$ mice (new Figure S2 of Supporting Information). We now also indicate in the Results that "*Rcan1 targeting affected both isoforms and lack of expression was confirmed in aorta and heart (Fig S2 of Supporting Information).*" (page 6, lines 2-3 of the first paragraph)

3. Fig.1. The findings using Western Blot analysis of RCAN1 protein expression in human atherosclerosis need to be validated by performing RT-PCR of both isoforms in human samples.

Following this reviewer's suggestion, RT-PCR of *RCAN1-1* and *RCAN1-4* was performed in human samples. The results showed that both isoforms were induced in atherosclerotic tissues (new Figure 1 of Supporting Information) and are described in the Results section of the revised version (page 5, end of first paragraph): "*The protein expression differences were accompanied by correspondingly higher expression of RCAN1-1 and RCAN1-4 mRNA in atherosclerotic arteries (Fig S1 of Supporting Information).*" In addition, a new paragraph addressing this issue has been added to the Discussion (page 11, last paragraph).

4. Fig. 5. Additional experiments need to be performed to show gene expression of CD36. What was the rationale for only investigating CD36? Flow cytometry analysis and gene expression of other scavenger receptors e.g. SRA-1 need to be included. Furthermore, although the data suggest that deletion of RCAN-1 inhibits oxLDL uptake the effect of cholesterol efflux should also be examined in macrophages from ApoE-/- and ApoE/RACN1 DKO animals. This is important. If the authors are correct and RCAN-1 mediates its effect via regulation of CD36 this needs to be further validated. Therefore, additional experiments showing the effect of overexpression of RCAN-1 on both foam cell formation and scavenger receptor expression need to be performed either in bone marrow derived (or peritoneal macrophages) or indeed in a relevant macrophage cell line.

These issues are highly relevant and we thank the reviewer for bringing them up. The rationale for investigating only CD36 expression in macrophages was based on preliminary evidence showing similar expression of SR-A in the aortic arch of atherosclerotic *Apoe*$^{-/-}$ and *Apoe*$^{-/-}$*Rcan1*$^{-/-}$ mice and lower levels of CD36 in Apoe-/-Rcan1-/- mice. Since these data were obtained in a small number of animals, we did not mention them. We have now extended these studies to include more mice and the data are presented in new Figure 7D and new Figure S7A of Supporting Information. In addition, we have determined SR-A levels in peritoneal macrophages and found them to be almost identical in *Apoe*$^{-/-}$ and *Apoe*$^{-/-}$*Rcan1*$^{-/-}$ cells (new Figure S7B of Supporting Information). These data are described in the revised version of the manuscript (last paragraph of page 7 - beginning of page 8)

We have also performed additional experiments to assess cholesterol efflux in *Apoe*$^{-/-}$ and *Apoe*$^{-/-}$*Rcan1*$^{-/-}$ macrophages. These data, shown in new Figure 8A of Supporting Information, indicate that Rcan1 ablation has a modest effect on cholesterol efflux. In accordance with this modest effect, the levels of ABCA and ABCG cholesterol transporters were similar in the atherosclerotic aortic arch of *Apoe*$^{-/-}$ and *Apoe*$^{-/-}$*Rcan1*$^{-/-}$ mice (new Figure 8B of Supporting Information). These results are also described in Results (page 8, paragraph 2).

We have generated lentiviral vectors to express Rcan1-1 and Rcan1-4 and additional lentiviral vectors to knockdown each isoform. Unfortunately, the latter vectors failed to efficiently knock-down Rcan1 expression in macrophages. However, we succeeded expressing both isoforms in *Apoe*$^{-/-}$*Rcan1*$^{-/-}$ macrophages (new Figure 7A). We found that forced expression of Rcan1-1 plus Rcan1-4 in these cells increased CD36 levels (new Figure 7B) and oxLDL uptake (new Figures 7C-7D). These results are described (page 8, lines 5-10 of paragraph 1) and discussed (page 12, lines 9-14 of second paragraph) in the revised version of the manuscript.

5. The data presented in Fig 6A and 6B should be presented as a single graph so that direct comparisons between Apoe-/- and ApoE/RACN-1 DKO can be illustrated. In Fig. 6C is the assumption that the inhibition of macrophage spreading in ApoE/RACN-1 macrophages as a results of CD36 inhibition? Again, this needs to be validated in vitro where macrophage spreading in macrophages transfected with RCAN-1.

As suggested by this reviewer, we combined the graphs from old Fig 6A and 6B in new Figure 8C.

We did not assume that the inhibition of cell spreading in *Apoe*$^{-/-}$*Rcan1*$^{-/-}$ macrophages is the result of CD36 inhibition and, in fact, we did not mention it in the original version of the manuscript. However, since oxLDL trigger the spreading of *Apoe*$^{-/-}$ macrophages and we propose that Rcan1

mediates oxLDL-induced spreading by regulating CD36 expression, we consider very likely that the inhibition of macrophage spreading in *Apoe*$^{-/-}$*Rcan1*$^{-/-}$ macrophages is the result of CD36 inhibition. We have attempted to experimentally validate this hypothesis, but unfortunately, we have encountered insurmountable obstacles. The most efficient procedure for over-expressing Rcan1 in *Apoe*$^{-/-}$*Rcan1*$^{-/-}$ macrophages is lentivirus-based transduction and, as mentioned above, we have succeeded in expressing both Rcan1 isoforms in these cells. The insuperable impediment of these experiments comes from the fact that transduced macrophages have to be detached prior to their seeding for spreading assessment. Once the macrophages had been plated, we could not detach and plate them again because they died during the manipulation.

6. Additional data showing the differences in serum/plasma of Il-10 levels in the animals groups would support the IL-10 expression.

As requested, we attempted to measure IL-10 levels in the plasma of atherosclerotic *Apoe*$^{-/-}$ and *Apoe*$^{-/-}$*Rcan1*$^{-/-}$ mice, but the levels were below the detection limit in all cases. Instead, we measured *IL-10* mRNA levels in the aortic arch of these animals. Our results show that *IL-10* expression is significantly higher in the aortic arch of *Apoe*$^{-/-}$*Rcan1*$^{-/-}$ mice (new Figure S11 of Supporting Information). These data are described in the Results (page 9, lines 9-10 of paragraph 3).

As suggested by this reviewer, the increased expression of iNos in Rcan1 deficient peritoneal macrophages might seem conflicting with the increased expression of IL-10 and other anti-inflammatory markers in these cells. We have performed additional experiments to determine iNos expression in atherosclerotic lesions of Apoe-/- and Apoe-/-Rcan1-/- mice. These data, shown in new Figure S13 of Supporting Information, indicate that Rcan1 inactivation has no significant effect on iNos expression in vivo. These results are described in Results "*Apoe*$^{-/-}$*Rcan1*$^{-/-}$ *cells also expressed higher levels of iNos, a marker of pro-inflammatory M1 macrophages (Figure 9C), but iNos expression in atherosclerotic lesions of the aortic arch was almost identical in Apoe*$^{-/-}$ *and Apoe*$^{-/-}$*Rcan1*$^{-/-}$ *mice (Fig S13 of Supporting Information). It thus seems that Rcan1 ablation might contribute to iNos induction by thioglycolate ex vivo, but not to its induction by proatherogenic stimuli in vivo.*" (page 10, lines 2-6)

7. Discussion. The authors state that RCAN1 is induced in mouse atherosclerosis and promotes disease progression. The data presented does not directly show that increased RCAN-1 increases disease progression. Therefore, the aforementioned in vitro validation experiments will facilitate the authors to conclude that RCAN-1 alters macrophage function towards a pro-inflammatory phenotype.

Although the results added in the revised version of the manuscript further support our conclusions, we have reworded the statement as follows: "*Our results demonstrate that RCAN1 is induced in human and mouse atherosclerosis and strongly suggest that Rcan1 promotes disease progression.*" (page 11, lines 3-5).

8. The data suggests that RCAN1 may inhibit macrophage egress. Evidence for this in vivo would provide a significant contribution to the manuscript although in the absence of relevant models this may be difficult to achieve.

We have used a previously reported model based on thioglycolate-induced recruitment of macrophages into the peritoneal cavity and their egress from this cavity following inoculation of LPS. Using this model, we found that Rcan1 depletion facilitates egress of oxLDL-treated macrophage cells (please see Figure below). However, we agree with the reviewer that there are no satisfactory models (this on included) for investigating the *in vivo* contribution of Rcan1 to the inhbition of macrophage egress, and we therefore chose not to include these data in the manuscript.

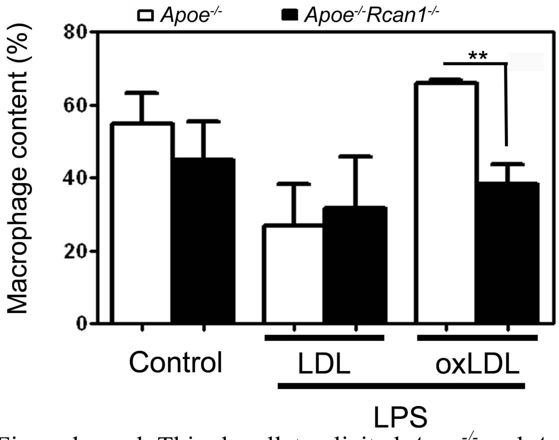

Figure legend: Thioglycollate-elicited *Apoe*$^{-/-}$ and *Apoe*$^{-/-}$*Rcan1*$^{-/-}$ peritoneal macrophages were isolated by lavage and counted 4h after mice were injected intraperitoneally with LPS. In some cases, mice were pretreated with 50 µg LDL or 50 µg oxLDL 1h prior to LPS injection. Data are the average of CD11b^{+} cells in the lavage (n=3; means±s.e.m; Student's t-test, p<0.01).

9. Supplementary Material. There is some degree of repetition between the supplementary methods and those in the main body of the manuscript for example the section on Laurdan GP microscopy (and indeed others) are reptitive and should be edited accordingly.

Following this reviewer's suggestion, we have eliminated unnecessarily repeated sections of the methods.

10. Supplementary Fig 1. Is there a significant decrease in body weight between ApoE-/- and ApoE/RACN1-/- before HFD? Perhaps this data would be better represented by showing % weight gain in the two animals groups.

Following this reviewer's suggestion, we have calculated % body weight gain of Apoe-/- and Apoe-/-Rcan1-/- mice new Figures S3 and S15 of Supporting Information. The data show that there is no significant difference in body weight gain between these genotypes.

11. Supplementary Figure 3. The authors indicate that macrophage motility is inhibited by oxLDL in a RCAN1 dependent manner. There are no statistical analysis to support this. Please include the p value for comparison between MCP-1 plus oxLDL in ApoE-/- and ApoE/RACN1-/-. Without this the data does not support the conclusion.

We inadvertently omitted the statistical analysis from this figure in the original submission. The analysis is now included in the new Figure S9 of Supporting Information.

12. Supplementary Figure 4. Is the difference in Arg1 expression statistically significant? This is referred to in the main body of the text and should be validated and p values included.

The p value was just above 0.05. However, we have increased the number of experiments and data are now significant (p = 0.011). The new Figure S12 of Supporting Information has therefore been modified accordingly.

Referee #2 (Remarks):

Mendez-Barbero et al report that RCAN1 promotes atherogenesis via polarization toward a pro-inflammatory macrophage phenotype, possibly due to the induction of the oxidized-LDL scavenger receptor CD36. After observing increased RCAN1 expression in multiple cellular lineages within

human atherosclerotic plaque, and corroborating this observation in ApoE-/- mice, they have defined the role of RCAN1 in atherogenesis using Rcan1 knockout mice crossed with ApoE-/-. Rcan1 loss is associated with a reduction in atheroma volume and complexity, along with reduced macrophage content. RCAN1 promoted oxidized-LDL uptake and mediated the inhibitory effect of oxidized-LDL upon macrophage migration in vitro. The authors suggest that the associated reduction in CD36 expression noted in Rcan1-/- macrophages explains many of their observations, although note that they cannot offer a mechanistic link between Rcan1 expression and CD36 expression. The importance of bone marrow derived cells to their observations is supported in a series of bone marrow transplantation studies with ApoE-/- recipients receiving donor material from ApoE-/- (control) or Rcan1-/-ApoE-/- mice.

The authors' work is interesting, appears well conducted and the manuscript is clearly written. Suggestions to improve the manuscript include:

1) Until tissue specific Rcan1-/- mice are available, the presented bone marrow transplantation studies are important to more strongly implicate monocyte/macrophage RCAN1. It is therefore important that the authors clarify whether Rcan1 alters the bone marrow reconstitution of recipients, since this may also alter their findings. For example, RCAN1 has been suggested to stimulate GSK3b expression in some tissues (FEBS J. 2006 May;273(10):2100-9); GSK3b is also important in modulating hematopoietic stem/progenitor cell function (e.g. J Clin Invest. 2013 Apr 1;123(4):1705-17) so may influence the nature of bone marrow reconstitution.

We thank this reviewer for his/her valuable assessment of our manuscript and helpful suggestions. The reviewer brings up an important question and although we had not observed significant differences in blood cell populations of *Apoe*$^{-/-}$ and *Apoe*$^{-/-}$*Rcan1*$^{-/-}$ mice, we carried out additional experiments to address this issue. We reconstituted the BM of irradiated Apoe-/- mice with cells from *Apoe*$^{-/-}$ or from *Apoe*$^{-/-}$*Rcan1*$^{-/-}$ donors and analyzed Gsk3ß expression and blood cell populations in recipient mice 4 weeks after transplantation. Gsk3ß expression was not significantly lower in DKO cells, and blood cell populations in mice recipients of *Apoe*$^{-/-}$ or *Apoe*$^{-/-}$*Rcan1*$^{-/-}$ cells were indistinguishable (new Fig 13 of Supporting Information). These data are presented in the Results section of the revised version (page 10, paragraph 2).

2) Whilst the authors do not offer a mechanistic link between RCAN1 and CD36 expression, short term knockdown or over-expression of Rcan1 in macrophages may provide further support of a temporal relationship between altered expression of Rcan1 and CD36. Such studies may also allow further probing of the mechanistic link with CD36.

The reviewer raises an important issue that we have also addressed in response to remark 4 of Reviewer #1. We have generated lentiviral vectors to express Rcan1-1 and Rcan1-4 and additional lentiviral vectors to knockdown each isoform. Unfortunately, the latter vectors failed to efficiently knock-down Rcan1 expression in macrophages. However, we succeeded in coexpressing both isoforms in *Apoe*$^{-/-}$*Rcan1*$^{-/-}$ macrophages (new Figure 7A). We found that forced expression of Rcan1-1 plus Rcan1-4 in these cells increased CD36 levels (new Figure 7B) and oxLDL uptake (new Figures 7C-7D). These results are described in the revised version of the manuscript (page 8, lines 5-10 of paragraph 1). A new sentence has also been added to the Discussion: "*This conclusion is supported by our findings that CD36, a scavenger receptor involved in the uptake of modified forms of LDL and foam-cell formation (Febbraio et al, 2000), is downregulated in Rcan1 deficient macrophages, while re-expression of Rcan1-1 and Rcan1-4 in these cells increased CD36 expression and foam-cell formation.*" (page 12, lines 7-14 of paragraph 2).

The reviewer brings up another important question. Although Rcan1 targeting generates less-advanced atherosclerotic plaques, these plaques could potentially be unstable. To address this issue, we have further characterized plaque structure and composition by measuring fibrous cap size and thickness (new Figure 5A and new Figure 4 of Supporting information), collagen and lipid content (new Figures 5B-5C), metalloprotease expression (new Figure 5 of Supporting Information) and intraplaque hemorrhage (new Figure 6 of Supporting Information). In addition, plaque stability score was calculated and is shown in the new Figure 5D. These new data, which are described in the

Results (page 7, first paragraph), show that Rcan1 inactivation increases fibrous cap area and thickness, decreases lipid content, does not affect collagen content or MMP expression, and does not promote plaque hemorrhage. We therefore conclude "*These data, together with the lower macrophage content of Rcan1-deficient plaques, thus indicate that Rcan1 targeting does not induce characteristics of unstable atherosclerotic plaques, and suggest instead that Rcan1 inactivation might increase plaque stability*". (page 7, end of first paragraph). We also state in the Discussion that "*While most lesions of Apoe$^{-/-}$ mice were advanced (grade IV), Apoe$^{-/-}$Rcan1$^{-/-}$ mice exhibited a greater number of early lesions (grade I) without showing any evidence of increased plaque instability.*" (page 12, lines 10-11 of first paragraph).

2nd Editorial Decision 03 September 2013

Please find enclosed the final reports on your manuscript. We are pleased to inform you that your manuscript is accepted for publication and is being sent to our publisher to be included in the next available issue of EMBO Molecular Medicine.

Congratulations on your interesting work,

***** Reviewer's comments *****

Referee #1 (Comments on Novelty/Model System):

In the revised manuscript the authors have provided additional evidence for the role of RCAN-1 in atherosclerosis and have adequately justified the use of the model system. Indeed the manuscript now provides convincing evidence for a causal role of hematopoietic RCAN-1 in atherosclerosis

Referee #1 (Remarks):

The authors have carefully and comprehensively addressed all previous comments and suggestions. The additional experiments investigating mRNA expression in human plaques, cholesterol efflux in RCAN-1 deficient macrophages and the use of lentiviral expression vectors have significantly enhanced the manuscript and support the conclusions.

Referee #2 (Remarks):

I note the extensive additional experiments and subsequent revisions to the original manuscript of MÈndez-Barbero et al. With specific regard to my own comments, I feel that the manuscript now sufficiently addresses these, and provides stronger mechanistic insights, along with greater translational relevance.
