# Supplementary material for: A major role for RCAN1 in atherosclerosis progression
Source: EMBO Mol Med. 2013 Oct 15;5(12):1901–17. doi: 10.1002/emmm.201302842 (PMC3914525; doi:10.1002/emmm.201302842)
Supplement: Supplementary file 2 [file emmm0005-1901-sd2.pdf]

SUPPORTING INFORMATION

Table of contents

Detailed Materials and Methods	Pages 1 - 9
Supporting Information References	Page 10
Supporting Information Figures	Pages 11 - 24

DETAILED MATERIALS AND METHODS

Animal procedures

Animal studies were approved by the local ethics committee, and all animal procedures conformed to EU Directive 2010/63EU and Recommendation 2007/526/EC regarding the protection of animals used for experimental and other scientific purposes, enforced in Spanish law under Real Decreto 1201/2005. C57BL/6J and *Apoe*$^{-/-}$ mice were obtained from Charles Rivers (JAX mice stock # 000664 and 002052, respectively). *Rcan1*$^{-/-}$ mice (mixed C57BL/6 and 129P2/OlaHsd background)(Porta et al, 2007), in which both *Rcan1-1* and *Rcan1-4* are targeted simultaneously, were backcrossed through >6 generations on a C57BL/6J background (Esteban et al, 2011) and subsequently crossed with *Apoe*$^{-/-}$ mice to create double-knockout *Apoe*$^{-/-}$*Rcan*$^{-/-}$ mice (Esteban et al, 2011). All mice were genotyped by PCR of tail samples. To accelerate atherosclerosis, 3-month-old mice were fed a HFD (10.8% total fat, 0.75% cholesterol; S4892-E010, Ssniff, Germany) for 6 wks. All experiments compared *Apoe*$^{-/-}$ mice to *Apoe*$^{-/-}$*Rcan*$^{-/-}$ littermates.

BM transplantation was performed as previously described (Esteban et al, 2011). Briefly, 2-month-old *Apoe*$^{-/-}$*Rcan*$^{+/+}$ mice were lethally irradiated (9 Gy) and transplanted through the tail vein with BM-derived cells (10^7) obtained from tibias and femurs of euthanized donor *Apoe*$^{-/-}$*Rcan*$^{-/-}$ or *Apoe*$^{-/-}$*Rcan*$^{+/+}$ mice. After 4 wk on chow diet, transplanted mice were placed on the HFD for 6 wk.

Whole blood was extracted postmortem by cardiac puncture and serum was obtained. Plasma concentrations of free cholesterol, total cholesterol, LDL-cholesterol, HDL-cholesterol and triglycerides were measured enzymatically using Kinetic colorimetric kits (Spinreact; Spain).

Circulating blood cells and platelets were counted and identified using a Petra Multi Link automated cell counter (Horiba). To count subpopulations of lymphocytes, erythrocytes were lysed in peripheral blood with red blood cell lysis buffer (1.5 M ammonium chloride, 0.1 M potassium bicarbonate, 0.1 M EDTA) for 3 min on ice. The reaction was stopped with PBS 1% BSA and cells were incubated with CD45 PerCP-Cy5.5 (BDPharmigen 550994), B220 Biotin (BDPharmigen 559971), CD3 AlexaFluor488 (BD Pharmigen 557666), CD4 Pe (BD Pharmigen 553653) and CD8 AlexaFluor647 (BD Phramigen 557682) for 30 min on ice. Streptavidin 405 (Invitrogen, S-32351) was used as secondary antibody. Cells were counted by flow cytometry using a BDFACS Canto II flow cytometer (BD Biosciences) and data were analyzed FlowJo software (Tree Star).

Human samples

Human coronary arteries and internal mammary arteries were collected from patients undergoing heart transplant and coronary artery bypass-graft surgery, respectively, at the Hospital de la Santa Creu i Sant Pau (Barcelona, Spain). Atherosclerotic and non-atherosclerotic coronary arteries were from coronary artery disease (CAD) and non-CAD patients, respectively. The studies were approved by the Ethics Committee and were conducted in accordance with the Helsinki Declaration.

Histological analysis and immunostaining

Mouse hearts and aortas were perfused with PBS, removed, fixed in 4% paraformaldehyde for 24 h, embedded in paraffin and prepared in 5-μm transverse sections for immunostaining or staining with H&E or Masson's Trichrome. Alternatively, mouse hearts were fixed in 10% paraformaldehyde for 24 h at 4ºC, incubated 24h in PBS supplemented with 30% sucrose and embedded in OCT and cryopreserved at -70ºC. Cryocut cross-sections (5 μm) of heart tissue were then prepared.

Deparaffinized sections were rehydrated and antigens retrieved in 10 mM citrate buffer pH 6.0 (95ºC, 20 min). Samples were blocked for 30 min with either 10% goat serum or 10% horse serum (for immunohistochemistry) or with 10% horse serum plus 2% BSA (for immunofluorescence), all in PBS. Cryocut cross-sections were stained with Oil Red (0.5% in isopropanol) or Masson's thricrome stain. Paraffin cross-sections were stained with hematoxilin-eosin or Masson's thricrome stain or were processed for immunohistochemistry or immunofluorescence. Samples were stained with rabbit anti-Rcan1 (D6694; Sigma-

Aldrich), alkaline phosphatase-conjugated mouse anti-smooth muscle actin (SMA) (A5691; Sigma-Aldrich), mouse anti-Mac3 (sc-1991; Santa Cruz Biotechnology), biotinylated lectin (L2140, Sigma-Aldrich), anti-Mrc1 (MCA2235GA; Serotec), anti-Ter119 (553672; BD), and biotinylated anti-IL-10 (Diaclone). For Rcan1 and Mac3 inmunohistochemistry, color was developed with DAB (Vector Laboratories) and sections were then counterstained with hematoxilin, dehydrated, and mounted in DPX (Fluka). For SMA, color was developed with Fast-Red Alkaline Phosphatase Substrate Tablets (Sigma-Aldrich).

Images were acquired at RT using a Leica DM2500 microscope with 10x, 20x or 40x HCX PL Fluotar objective lenses and Leica Application Suite V3.5.0 acquisition software. Secondary antibodies for immunofluorescence were Alexa Fluor Cy3-conjugated goat anti-rabbit (excitation at 555 nm, emission at 500-610 nm), Alexa Fluor 488-conjugated chicken anti-mouse (excitation at 488 nm, emission at 505-600 nm), Alexa Fluor 647 chicken anti-rat (Invitrogen; excitation at 633 nm, emission at LP640 and pseudocolored in red) and streptavidin-Alexa Fluor 647 (Invitrogen; excitation at 633 nm, emission at LP640 and pseudocolored in green). Nuclei were stained with DAPI (excitation at 405 nm, emission at 420-475 nm). Immunofluorescence stainings were mounted in Citifluor AF4 mounting medium (Aname) and images (1024 x 1024 pixels; 8-bit) were acquired at RT using an inverted confocal microscope (LSM700; Carl Zeiss) with 63x Plan-Apochromat oil immersion objectives. Images were analyzed using ImageJ (http://rsbweb.nih.gov/ij/index.html) or MetaMorf (Molecular Devices; Sunnyvale, California) and were processed for presentation with Zen 2009 Light Edition (Carl Zeiss) and Adobe Photoshop.

Cell procedures

VSMCs were extracted from abdominal and thoracic aortas as described (Esteban et al, 2011) and cultured in Dulbecco's modified Eagle medium (DMEM, GIBCO-Invitrogen) containing 10% fetal bovine serum (FBS; Lonza), L-glutamine and antibiotics (100 units/ml penicillin and 100 μg/ml streptomycin). Mouse lung endothelial cells (MLECs) were obtained from mouse lungs digested with 0.2% collagenase P (Roche) for 1 h at 37°C, and further disaggregated to produce single-cell suspensions. The mixed population obtained was subjected to negative selection with magnetic beads (Dynal) coated with anti-CD16 (BD Biosciences) followed by positive selection with magnetic beads coated with anti-ICAM-2 (BD Biosciences). This procedure resulted in a >90% pure population of endothelial cells.

MLECs were grown on 0.5% gelatin, 100mg/ml collagen I (Sigma) in DMEM F-12 (Biomlutaker BE. 12-719F) containing 20% FBS, heparin (100mg/ml), EGCF (5mg/ml), glutamine (Sigma), and antibiotics (100 units/ml penicillin and 100 µg/ml streptomycin). Before stimulation, cells were rendered quiescent by 48 h culture in DMEM without FBS (VSMCs) or overnight culture with 0.5% FBS (MLECs).

Peritoneal macrophages were collected from mice by peritoneal lavage 4 days after intraperitoneal injection of 3% (wt/vol) thioglycolate. Cells were used for migration experiments or were cultured 24-48 h in AlphaMEM (Lonza) supplemented with 10% FBS, L-glutamine and antibiotics and stimulated for 4-24 h in medium containing 2% FBS and 50μg/ml LDL (Invitrogen) or 50μg/ml oxLDL (Biomedical Technologies).

Flow cytometry.

Peritoneal macrophages were cultured for 72h and then suspended in PBS containing 1% BSA, pre-incubated with Mouse BD Fc Block (BD Pharmingen, 553141) and costained for 30 min at 4ºC with anti-Mouse F4/80 Antigen PE-Cyanine7 (eBioscience, 25-4801-82) and rabbit anti-CD36 (Abcam, ab78054) or anti-SR-A (Santa Cruz, sc-20444). Alexa Fluor 647-conjugated goat anti-rabbit (Invitrogen, A-21443) and Alexa Fluor 647-conjugated chicken anti-goat (Invitrogen, A-21469) were used as secondary antibodies. Cells were counted by flow cytometry using a BD FACS Canto II flow cytometer (BD Biosciences) and data were analyzed with FlowJo software (Tree Star).

Western blot analysis

Human specimens and mouse aortic samples for western blotting were snap-frozen in liquid nitrogen and stored at -80ºC. Human protein extracts were obtained using an ice-cold lysis buffer containing 50 mM Tris-HCl pH 7.5, 1% (w/v) Triton X-100, 150 mM NaCl and 1 mM DTT, supplemented with phosphatase and protease inhibitors (Roche) (Fuster et al, 2011). Mouse tissue protein extracts were obtained using ice-cold lysis buffer containing 10 mM Tris-HCl pH 7.5, 1% Triton X-100, 1% sodium deoxicholate, 0.1% SDS, 150 mM NaCl and 5mM EDTA, supplemented with 1 μM dithiothreitol, 1 mM phenylmethylsulfonyl fluoride, 100 μM benzamidine, 1 μg/ml pepstatin and 1μg/ml aprotinin.

After stimulation of primary cultures, cells were washed with ice-cold PBS and lysed with 20 mM Hepes pH 7.6 containing 1% Triton X-100, 0.4 M NaCl, 1 mM EDTA, 3 mM EGTA, 1 μM dithiothreitol, 1 mM phenylmethylsulfonyl fluoride, 100 μM benzamidine, 1 μg/ml pepstatin and 1μg/ml aprotinin.

Proteins were separated under reducing conditions on SDS-polyacrylamide gels and transferred to nitrocellulose membranes. Protein detection was performed with anti-Rcan1 (D6694; Sigma-Aldrich), anti-α-actin (M0851, Dako), anti-Gsk3ß (9315; Cell Signaling), anti-PSF (P2860; Sigma-Aldrich) anti-alpha-tubulin (T6074; Sigma-Aldrich) and HRP-conjugated secondary antibodies (Pierce). Immunocomplexes were detected with enhanced chemiluminescence (ECL) detection reagent (Millipore).

Atherosclerotic lesion analysis

Hearts from euthanized mice were perfused through the left ventricle with PBS. After fixing in 4% paraformaldehyde overnight at 4°, the aortas were thoroughly cleaned under a dissecting microscope to remove all adventitial fat and connective tissue. Aortas were whole-mount stained with 0.2% Oil Red O (Sigma O0625) in 80% methanol, opened longitudinally and pinned to black wax to expose the entire luminal surface. Images were acquired using an Olympus SZX10 stereomicroscope (Olympus, Germany) coupled to an Olympus UC30 digital color camera (Olympus, Germany). The planimetric area of atherosclerotic plaques was measured in pixels using ImageJ and converted to mm^2.

Migration assays

Migration of peritoneal macrophages was measured in a modified Boyden chamber using Transwell inserts with a 5 μm-pore membrane (Costar). Medium containing 2% FBS and 100ng/ml MCP-1 (RD Systems) was placed in the lower wells in some cases. Cells (1-2 x 10^5 per well) in AlphaMEM supplemented with 0.1% BSA were loaded into the migration chamber with 50μg/ml of lipoprotein, (LDL, oxLDL or acLDL). After allowing cell migration for 18 h, cells were removed from the upper side of membranes, and nuclei of migrated cells on the lower side of the membrane were stained with Hoechst. The number of migrated cells was counted on fluorescence microscopy photographs of 10 randomly-selected fields. Experiments were performed in triplicate.

For wound healing assays, a single scrape wound was made on monolayers of peritoneal macrophages seeded 3 days before on 35-mm-diameter plates. After washing with PBS, cells were incubated with 2% FBS plus 100ng/ml MCP-1 with or without 50ug/ml oxLDL. The number of cells that migrated into the denuded area was counted using ImageJ. Macrophage motility was monitored by time-lapse videomicroscopy.

Spreading assays

Peritoneal macrophages (10^4 cells) were placed on serum-coated slides and allowed to attach to the surface at 37ºC for 20 minutes. After stimulation of macrophages with 50μg/ml oxLDL for the indicated times, cells were fixed in 4% paraformaldheyde for 15 minutes and stained with fluorescein-conjugated phalloidin. Images were taken using an inverted confocal microscope (LSM700; Carl Zeiss) fitted with a 63x Plan-Apochromat oil immersion objective. Cell perimeter and surface was measured with cell profile software and spreading was determined according to the formula Spreading = perimeter^2/(4 x π x area).

Foam-cell formation.

Peritoneal macrophages were plated on coverslips, incubated with 50μg/ml LDL or oxLDL for 24h, fixed in 4% paraformaldehyde for 15 minutes and stained with Oil red O and counterstained with hematoxylin.

Laurdan GP microscopy.

Laurdan GP microscopy has been described previously (Bagatolli et al, 2003; Sanchez et al, 2007). Peritoneal macrophages were cultured in the presence of 1 μM Laurdan (Molecular Probes, Grand Island, NY) for 30 minutes in serum-free medium, using DMSO as vehicle. Laurdan fluorescence was excited with a mode-locked titanium-sapphire laser (Spectra-Physics Mai Tai DS) set at 780 nm and its emission collected at 445-465 nm and 474-514 nm. GP images (512x512, 32 bits) were obtained with an ALBA microscope (ISS, Illinois) equipped with a 63x water objective (1.2 NA) and analyzed using Image-J software (NIH Image, National Institute of Health, Bethesda, MD). Laurdan GP was calculated by using a G factor of DMSO-dissolved Laurdan = 0.57 (Bagatolli et al, 2003) and applying the following equation to every pixel:

$$Laurdan\ GP = \frac{I_{(425-465nm)} - G \times I_{(476-514nm)}}{I_{(425-465nm)} + G \times I_{(476-514nm)}}$$

where $I_{425\text{-}465}$ and $I_{476\text{-}514}$ are the emission intensities at 445-465 and 474-514 nm respectively.

Cholesterol accumulation and efflux

Peritoneal macrophages were plated at 5 × 10^5 cells/well in 24-well plates. After 24 h, triplicate wells were cultured in Alpha MEM with 10% human lipoprotein deficient serum, in the presence of 2 μCi/ml of ^{3}H-cholesterol (Perkin Elmer) and 50 μg/ml of acLDL (Biomedical Technologies) for 24h (cholesterol accumulation) or 32h (cholesterol efflux). ^{3}H-cholesterol accumulation was measured by scintillation counting after extraction of cellular lipids with hexane/isopropanol (2:1). For efflux assessment, cells were extensively washed in PBS, and then incubated in Alpha-MEM plus either 100 μg/ml HDL (Biomedical Technologies) or 0.1 % BSA for 24 h. Radioactivity was measured by scintillation counting in the medium and in cellular lipids after extraction with hexane/isopropanol (2:1). HDL-induced cholesterol efflux was calculated using the following equation:

$$\%\,efflux = \left(\frac{cpm\ in\ medium}{cpm\ in\ medium + cpm\ in\ cells}\cdot 100\right)^{HDL} - \left(\frac{cpm\ in\ medium}{cpm\ in\ medium + cpm\ in\ cells}\cdot 100\right)^{BSA}$$

Lentiviral production and infection

HA-tagged *RCAN1-1* and *RCAN1-4* cDNA were excised from pHA-CALP1L and pHA-CALP1S (a generous gift of Dr. S. de la Luna) and cloned into the lentiviral vector pHRSIN-IRES-GFP to generate pHRSIN-HA-RCAN1-1-IRES-GFP and pHRSIN-HA-RCAN1-4-IRES-GFP.

Lentiviruses expressing HA-RCAN1-1-IRES-GFP and HA-RCAN1-4-IRES-GFP were obtained by transient calcium phosphate transfection of HEK-293 cells, employing a three plasmid HIV-derived and VSV pseudotyped lentiviral system kindly provided by M. K. Collins (University College London, UK). The supernatant containing the lentiviral particles was collected 48 h after removal of the calcium phosphate precipitate, filtered through a 45 μM PVDF membrane (Steriflip, Millipore) and ultracentrifuged for 2 h at 26,000 rpm, 4ºC (Ultraclear Tubes, SW28 rotor and Optima L-100 XP Ultracentrifuge; Beckman). Viruses were resuspended in cold sterile DMEM and titrated in Jurkat cells by flow cytometry. Infection efficiency (GFP-expressing cells) and cell death (propidium iodide staining) were

monitored by flow cytometry. Similar infection efficiencies were obtained with the different constructs and across experiments.

Peritoneal macrophages (10^6 cells) were plated, washed with PBS 24h later and infected with lentiviruses encoding IRES-GFP (Mock) or RCAN1-1-IRES-GFP plus RCAN1-4-IRES-GFP. After 24h, medium was replaced with AlphaMEM supplemented with 10% FBS. CD36 expression was assessed 48h later in GFP-f4/80 double-positive cells by flow cytometry. For foam-cell formation assays, culture medium was replaced after 24h of infection with AlphaMEM supplemented with 10% human lipoprotein-deficient serum. Cells were washed after 48h cells and then incubated with 50 μg/ml oxLDL for 24h. oxLDL uptake was determined by Laurdan GP microscopy.

Antigen presentation

Macrophages were cultured in 35-mm-diameter plates in the presence of 2 mg/ml OVA for 2 h. After washing several times with PBS, macrophages were cultured for 5 h in regular culture medium and β-galactosidase-expressing B3Z T cells (2×10^6) were added overnight. Antigen presentation was quantified by assessing the hydrolysis of chlorophenol red-β-D-galactopyranoside (CPRG) (Calbiochem) with a spectrophotometer (O.D. 595-655)

Phagocytosis

Macrophages (3×10^5) were cultured overnight on glass coverslips. Non-adherent cells were removed and macrophages were cultured without FBS for 2 h. Sheep red blood cells were opsonized with rabbit IgG or left untreated, and then added to the macrophage culture for 15 min. Cells were fixed, permeabilized and stained with phalloidin-TxRed (Invitrogen) and goat anti-rabbit 647 to detect phagocytosed particles. Internalized red blood cells were counted under a microscope.

Quantitative PCR analysis

Total RNA was isolated with TRIZOL (Life Technologies), DNase-treated and reverse-transcribed at 37ºC for 50 min in a 20μl reaction mix containing 200U Moloney murine leukemia virus (MMLV) reverse transcriptase (Life Technologies), 100ng random primers, and 40U RNase Inhibitor (Life Technologies). Reactions were terminated by heating at 95ºC for 5 min. Real-time quantitative RT-PCR was performed using Prime Time qPCR assay (Integrated DNA Technologies) specific for human *GAPDH* (Hs.PT.39a.22214836), TaqMan

Gene Expression assays (Life Technologies) specific for human *RCAN1-1* (Hs01120956_m1) and mouse *Rcan1* (Mm00627762_m1) and *Hprt1* (Mm00446968_m1) and a custom TaqMan Gene Expression assay specific for *RCAN1-4* (primers: GCAAACAGTGATATCTTCAGCGAAA, GTGATGTCCTTGTCATACGTCCTAA; probe: CAGGGCCAAATTT). SYBR Green was used for RT-PCR detection of *IL-10* (TGCTATGCTGCCTGCTCTTA, TCATTTCCGATAAGGCTTGG), *Mrc1* (ATGCCAAGTGGGAAAATCTG, TGTAGCAGTGGCCTGCATAG), *Arg1* (CTCCAAGCCAAAGTCCTTAGAG, AGGAGCTGTCATTAGGGACATC), *Mcp-1* (ACACCCTACAAACCGGAACC, AGCCTTCCTGTCATAGTATTCCT), *Abca1* (AGTGATAATCAAAGTCAAAGGCACAC, AGCAACTTGGCACTAGTAACTCTG), *Abcg1* (TTCATCGTCCTGGGCATCTT, CGGATTTTGTATCTGAGGACGAA), *SR-A* (TGAACGAGAGGATGCTGACTG, GGAGGGGCCATTTTTAGTGC), *Mmp2* (GCACCACCGAGGACTATGAC, ACTTGTTGCCCAGGAAAGTG), *Mmp9* (CAAATTCTTCTGGCGTGTGA, CGGTTGAAGCAAAGAAGGAG), *iNos* (CAGCTGGGCTGTACAAACCTT, CATTGGAAGTGAAGCGTTTCG) and m36B4 (GCGACCTGGAAGTCCAACTA, ATCTGCTGCATCTGCTTGG). Calculations were made from measurements of 3 replicates of each sample. The amount of target mRNA in samples was estimated by the 2CT relative quantification method using *GAPDH*, *Hprt1* or m36B4 for normalization.

CN phosphatase activity.

CN enzyme activity in macrophage extracts was analyzed with the Biomol Green Calcineurin Assay kit (Enzo Life Sciences) according to the manufacturer's instructions.

Statistical analysis.

All values are expressed as means ± SEM. Differences were evaluated using one-way or two-way analysis of variance (ANOVA) and Bonferroni's post-hoc test (experiments with ≥3 groups) or Student's t-test, as appropriate for the data. Statistical significance was assigned at $p<0.05$.

SUPPORTING INFORMATION REFERENCES

Bagatolli LA, Sanchez SA, Hazlett T, Gratton E (2003) Giant vesicles, Laurdan, and two-photon fluorescence microscopy: evidence of lipid lateral separation in bilayers. Methods Enzymol 360**:** 481-500

Esteban V, Mendez-Barbero N, Jimenez-Borreguero LJ, Roque M, Novensa L, Garcia-Redondo AB, Salaices M, Vila L, Arbones ML, Campanero MR et al (2011) Regulator of calcineurin 1 mediates pathological vascular wall remodeling. J Exp Med 208**:** 2125-2139

Fuster JJ, Gonzalez-Navarro H, Vinue A, Molina-Sanchez P, Andres-Manzano MJ, Nakayama KI, Nakayama K, Diez-Juan A, Bernad A, Rodriguez C et al (2011) Deficient p27 phosphorylation at serine 10 increases macrophage foam cell formation and aggravates atherosclerosis through a proliferation-independent mechanism. Arterioscler Thromb Vasc Biol 31**:** 2455-2463

Porta S, Serra SA, Huch M, Valverde MA, Llorens F, Estivill X, Arbones ML, Marti E (2007) RCAN1 (DSCR1) increases neuronal susceptibility to oxidative stress: a potential pathogenic process in neurodegeneration. Hum Mol Genet 16**:** 1039-1050

Sanchez SA, Tricerri MA, Gunther G, Gratton E (2007) Laurdan generalized polarization: from cuvette to microscope. In Modern Research and Educational Topics in Microscopy: applications in physical/chemical sciences, Mendez-Vilas A, Diaz J (eds) pp 1007-1014. Formatex

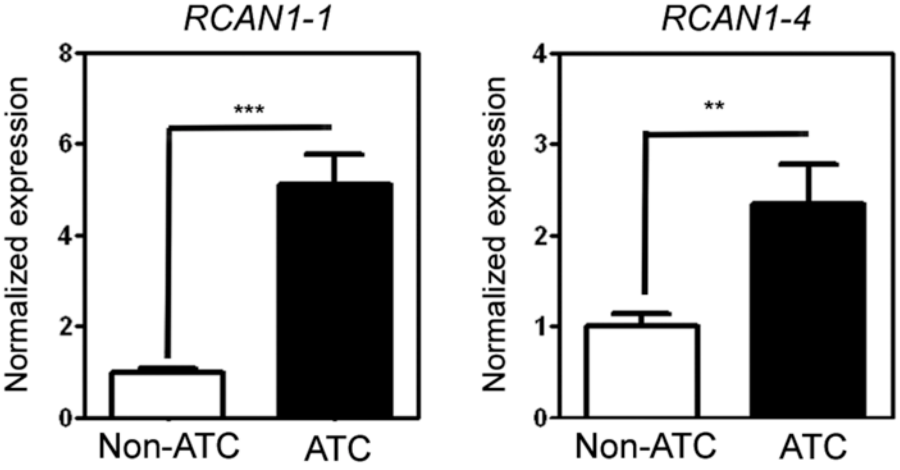

Figure S1. ***RCAN1*** **is induced in human atherosclerotic arteries.** Quantitative PCR analysis of *RCAN1-1* and *RCAN1-4* expression in atherosclerotic (ATC) and non-atherosclerotic (Non-ATC) human coronary arteries. mRNA amounts were normalized to *GAPDH* expression (means±s.e.m). Student's t-test, **p=0.008, ***p=0.0001.

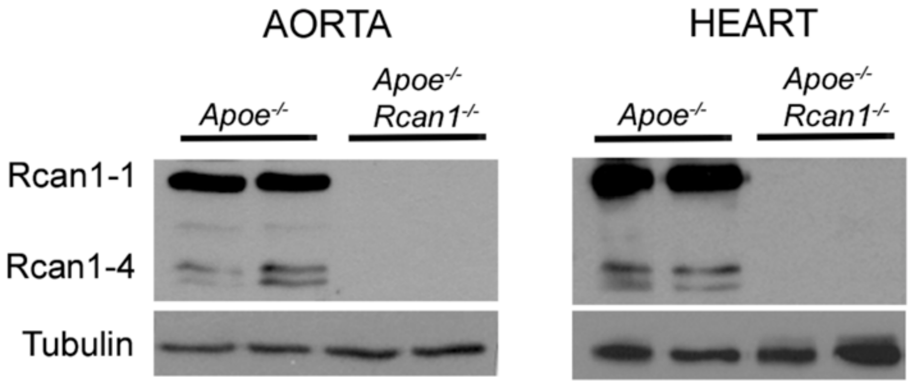

Figure S2. ***Rcan1*** **gene targeting ablates expression of Rcan1-1 and Rcan1-4.** Rcan1 immunoblot staining of aorta and heart tissue extracts from atherosclerotic *Apoe*$^{-/-}$ and *Apoe*$^{-/-}$*Rcan1*$^{-/-}$ mice (one mouse per lane). Tubulin shows similar protein loading. Data are representative of 5 mice per group.

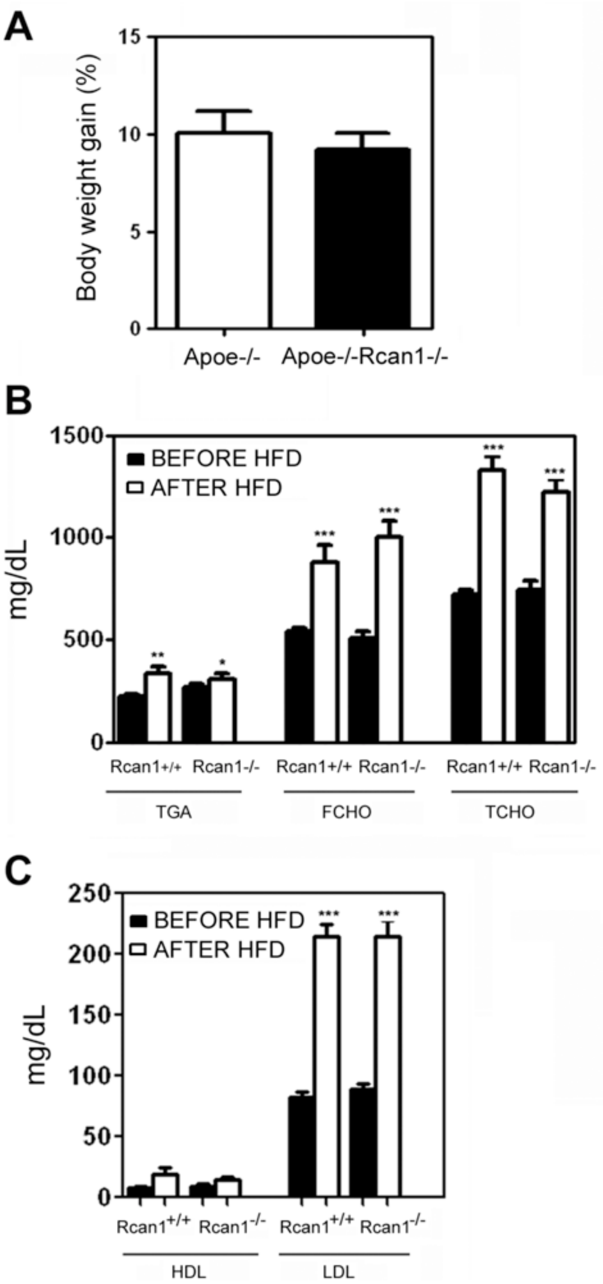

Figure S3. HFD-induced body weight increase and lipid profile changes are not mediated by Rcan1. (**A**) Body weight gain of *Apoe*$^{-/-}$ (Rcan1+/+; n=12) and *Apoe*$^{-/-}$ *Rcan1*$^{-/-}$ (Rcan1-/-; n=18) mice after 6 weeks on a HFD. (**B**) Serum concentration of triglyceride (TGA), free cholesterol (FCHO) and total cholesterol (TCHO) in these mice before and after diet (means±s.e.m). (**C**) Serum levels of high-density lipoproteins (HDL) and LDL in the same mice (means±s.e.m). One-way ANOVA, *p=0.014, **p=0.0036, ***p<0.00001.

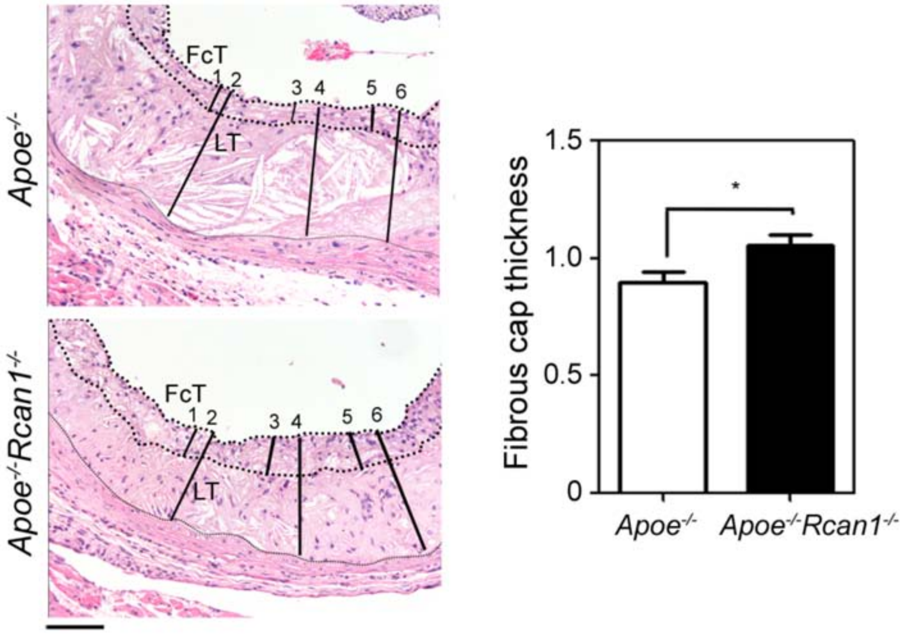

Figure S4. *Rcan1* gene targeting increases fibrous cap thickness. (**A**) Representative images of H&E staining of lesions in the aortic sinus of *Apoe*$^{-/-}$ (n=25 valves) and *Apoe*$^{-/-}$ *Rcan1*$^{-/-}$ mice (n=45 valves) fed a HFD for 6 wk. The average of three measurements of fibrous cap thickness (FcT) and lesion thickness (LT) taken in the center of each lesion was calculated. Odd and pair lines indicate the measurements of fibrous cap thickness and lesion thickness, respectively, taken in these particular lesions. Scale bar, 50 μm. (**B**) Quantification of fibrous cap thickness relative to lesion thickness and normalized (means$\pm$s.e.m; Student's t-test, *p=0.016).

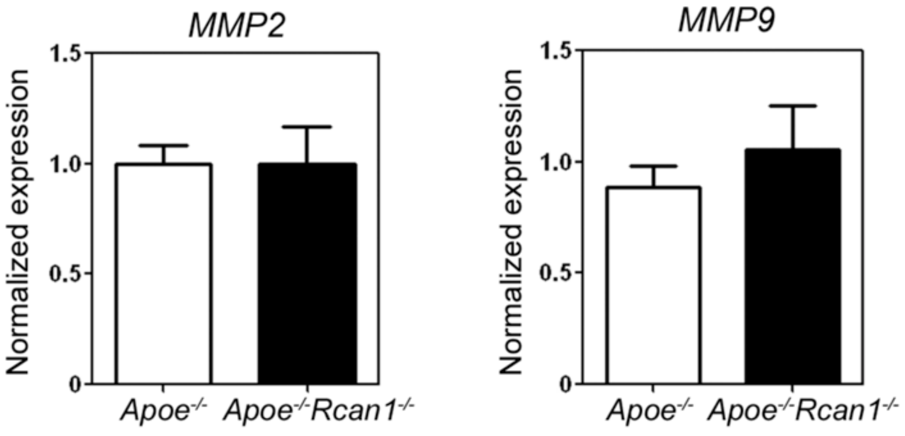

Figure S5. *Rcan1* gene targeting does not increase *MMP2* or *MMP9* expression. Quantitative PCR analysis of *MMP2* and *MMP9* expression in the aortic arch of atherosclerotic *Apoe*$^{-/-}$ (n=12) and *Apoe*$^{-/-}$*Rcan1*$^{-/-}$ mice (n=14) pooled from three independent experiments. mRNA amounts were normalized to *m36B4* expression (means$\pm$s.e.m). Student's t-test, non-significant.

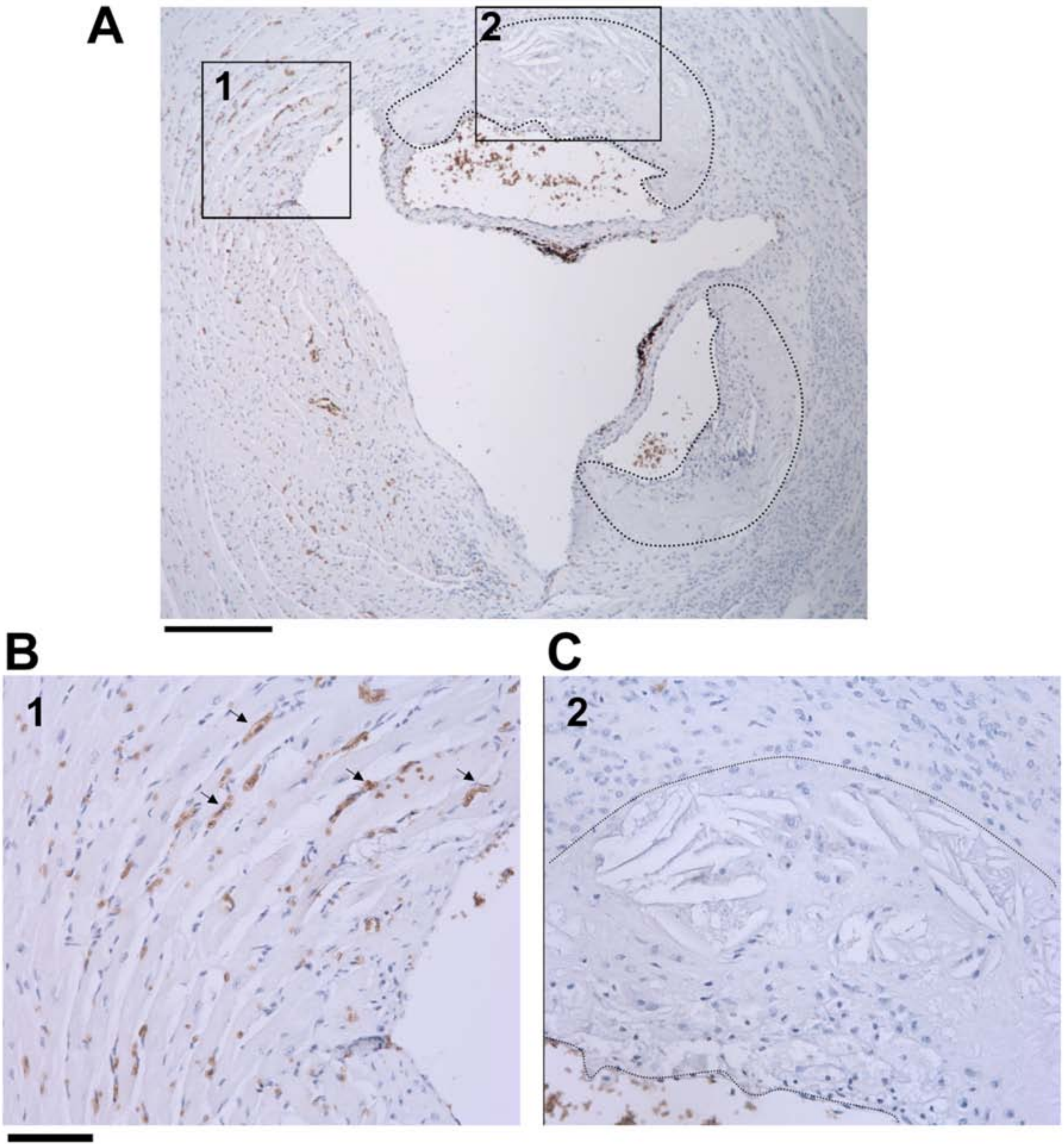

Figure S6. *Rcan1* gene targeting does not induce intraplaque hemorrhage. (**A**) Representative Ter-119 immunohistochemistry of the aortic sinus of an atherosclerotic *Apoe*$^{-/-}$*Rcan1*$^{-/-}$ mouse. Scale bar, 200 μm. (**B-C**) Enlargement of boxed regions 1 and 2. Erythrocytes (Ter-119^{+} cells) are found only in the heart tissue (B) and the lumen (bottom left corner of C). Arrows point to Ter-119^{+} cells. Scale bar, 50 μm. Images are representative of 12 aortic valves from 4 *Apoe*$^{-/-}$ mice and of 12 lesions from 4 *Apoe*$^{-/-}$*Rcan1*$^{-/-}$ mice.

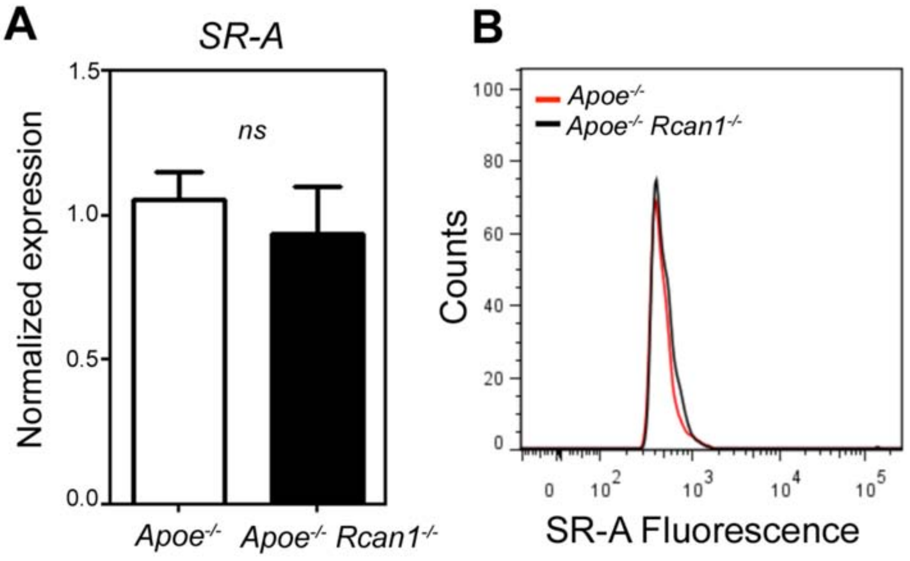

Figure S7. ***SR-A*** **expression is not affected by** ***Rcan1*** **gene targeting.** (**A**) Quantitative PCR analysis of *SR-A* expression in the aortic arch of atherosclerotic *Apoe*$^{-/-}$ (n=12) and *Apoe*$^{-/-}$*Rcan1*$^{-/-}$ mice (n=13) pooled from three independent experiments. mRNA amounts were normalized to *m36B4* expression (means±s.e.m). Student's t-test, non-significant (*ns*). (**B**) Representative flow cytometry fluorescence histograms of SR-A-stained macrophages of the indicated genotypes.

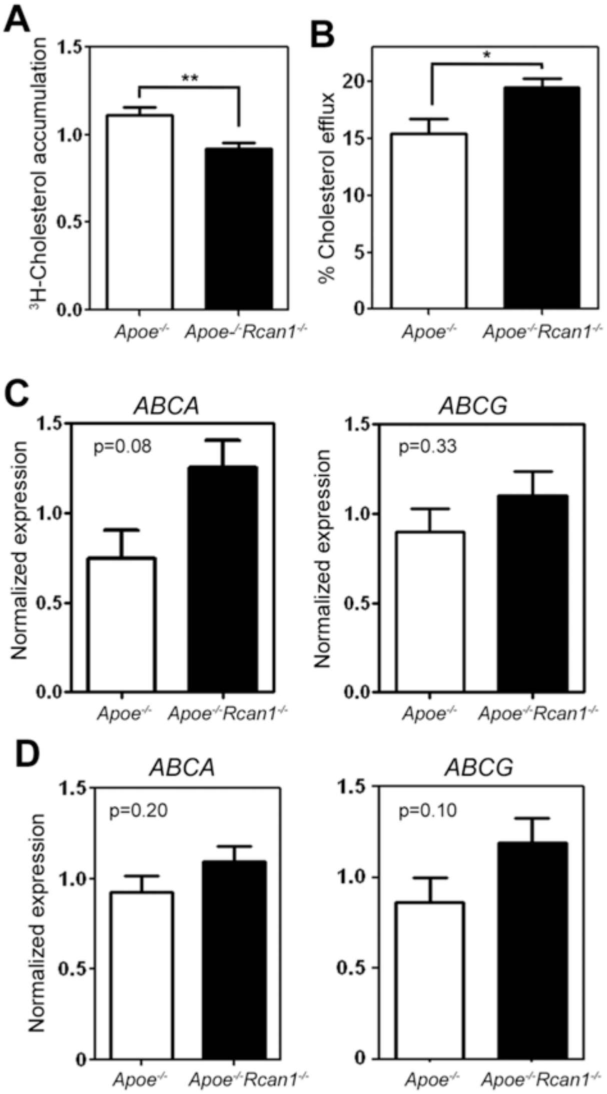

Figure S8. Rcan1 is a modest regulator of cholesterol efflux in macrophages. (**A**) ^{3}H-cholesterol accumulation in acLDL-loaded cultured peritoneal macrophages and (**B**) ^{3}H-cholesterol efflux in cultured peritoneal macrophages treated with 100 μg/ml HDL for 24h. Data are means$\pm$s.e.m (n=4). Student's t-test, *p=0.04, **p=0.005. Quantitative PCR analysis of *ABCA* and *ABCG* expression in (**C**) cultured peritoneal macrophages (n=4) and (**D**) the aortic arch of atherosclerotic *Apoe*$^{-/-}$ (n=12) and *Apoe*$^{-/-}$*Rcan1*$^{-/-}$ mice (n=13) pooled from three independent experiments. mRNA amounts were normalized to *m36B4* expression (means$\pm$s.e.m). Student's t-test, as indicated.

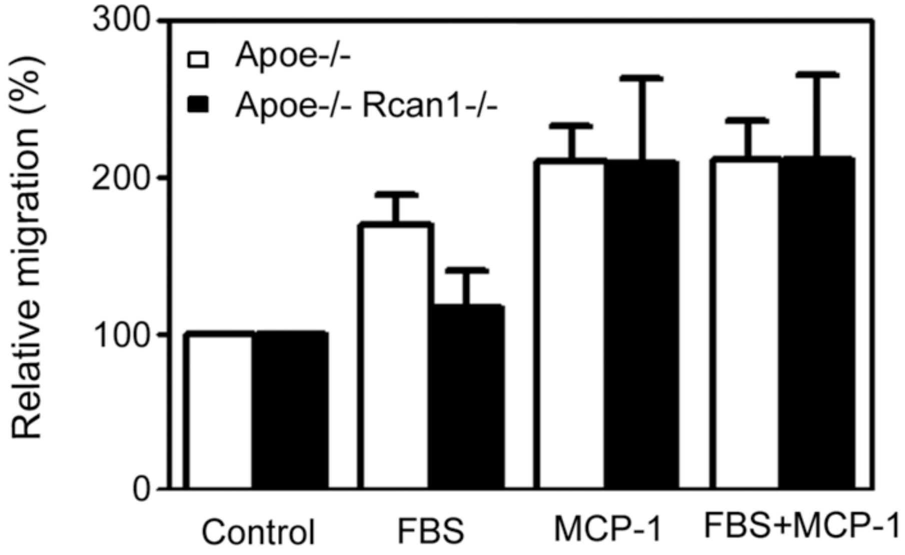

Figure S9. Rcan1 does not mediate macrophage chemotaxis. *Apoe*$^{-/-}$ and *Apoe*$^{-/-}$ *Rcan1*$^{-/-}$ macrophages were seeded on the upper surface of chemotaxis chambers containing medium alone (Control) or medium supplemented with FBS, MCP-1 or FBS plus MCP-1. After 18 h, non-migrating cells were removed from the upper filter surfaces and the filter was stained with Hoechst. Quantification of migrated macrophages in ten fields per condition. Data are shown relative to non-treated cells (mean ± s.e.m, n=4).

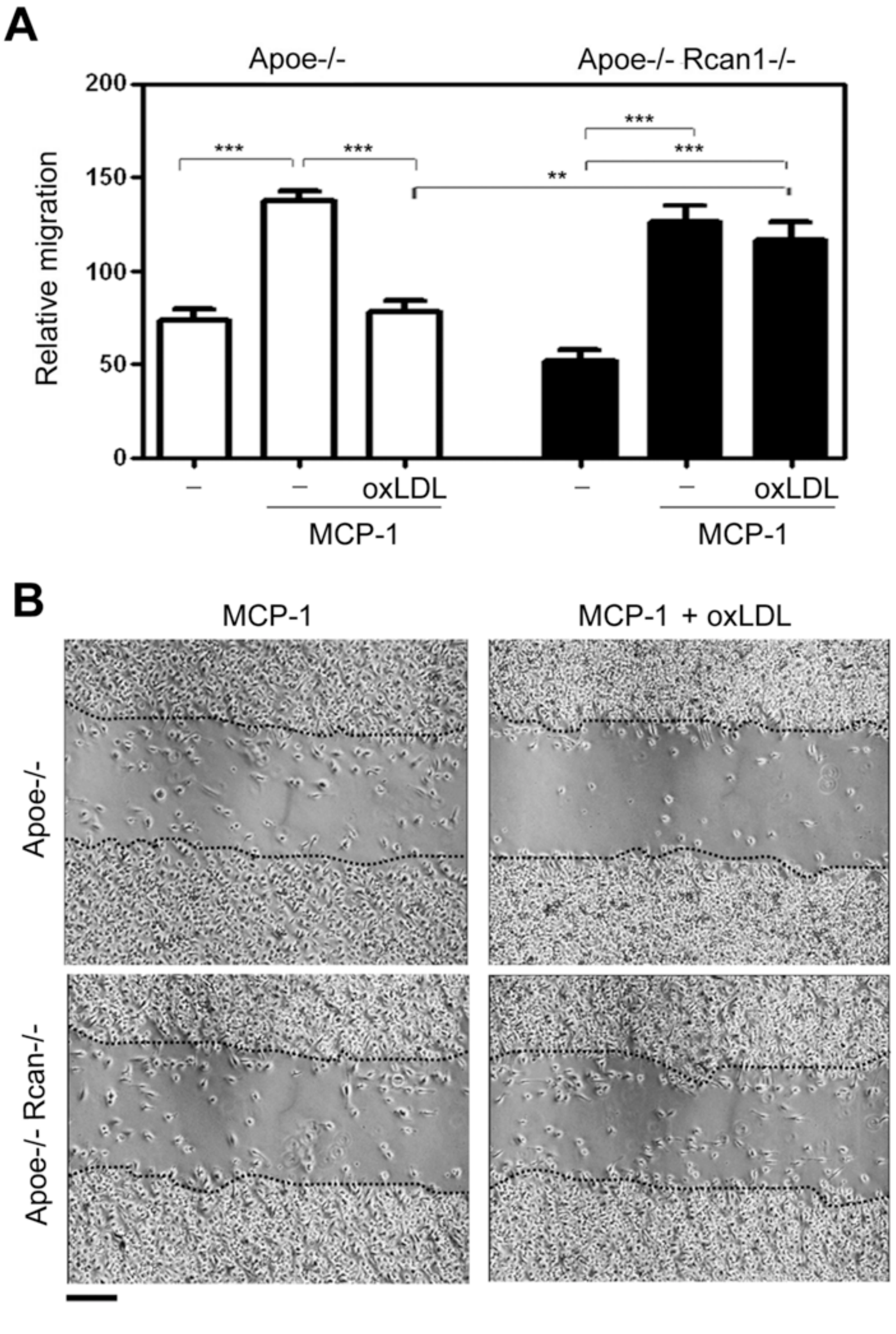

Figure S10. Macrophage motility is inhibited by oxLDL in an Rcan1-dependent manner. A single scrape wound was made on confluently plated *Apoe*$^{-/-}$ and *Apoe*$^{-/-}$ *Rcan1*$^{-/-}$ macrophages, and cells were then incubated in medium alone or medium supplemented with 100 ng/ml MCP-1 or 100 ng/ml MCP-1 plus 50 μg/ml oxLDL. Migration into the denuded area was monitored by photomicroscopy. (**A**) Quantification of migrated macrophages in ten fields per condition. Data show number of cells relative to the surface area of the scrape (mean ± s.e.m n=3; one-way ANOVA, **p=0.0037, ***p<0.0001). (**B**) Representative images of macrophages in scraped regions. Bar, 100 μm.

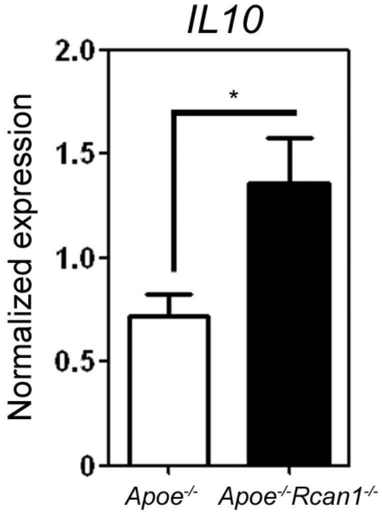

Figure S11. ***Rcan1*** **gene targeting increases IL10 gene expression in the aortic arch of atherosclerotic mice.** Quantitative PCR analysis of *IL10* expression in the aortic arch of atherosclerotic *Apoe*$^{-/-}$ (n=12) and *Apoe*$^{-/-}$*Rcan1*$^{-/-}$ mice (n=13) pooled from three independent experiments. mRNA amounts were normalized to *m36B4* expression (means±s.e.m). Student's t-test, *p=0.0108.

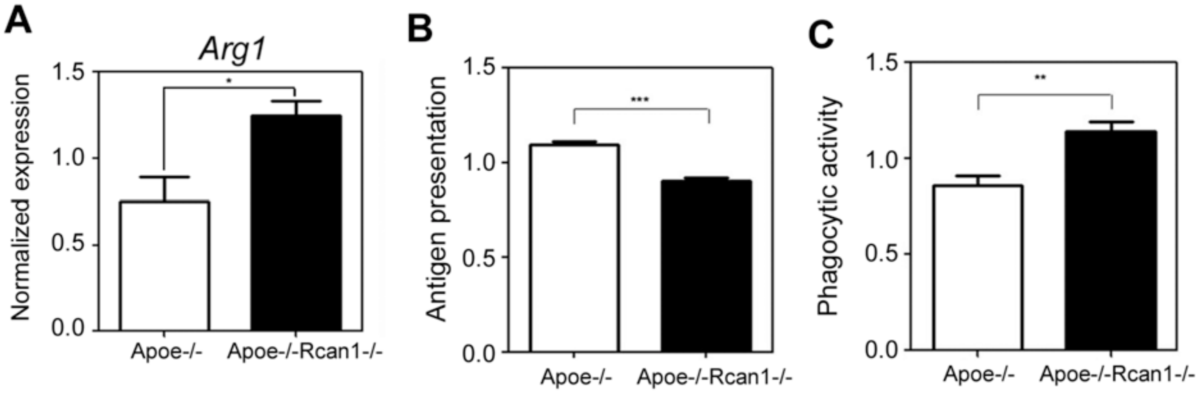

Figure S12. Rcan1 regulates ***Arg1*** **expression, antigen presentation and phagocytic activity in macrophages.** (**A**) Quantitative PCR analysis of *Arg-1* mRNA expression in *Apoe*$^{-/-}$ and *Apoe*$^{-/-}$*Rcan1*$^{-/-}$ peritoneal macrophages. mRNA amounts were normalized to *m36B4* expression (mean±s.e.m; n=7). (**B**) Antigen (ovalbumin) presentation by *Apoe*$^{-/-}$ and *Apoe*$^{-/-}$*Rcan1*$^{-/-}$ macrophages to B3Z T-cell hybridoma cells. Data are means±s.e.m (n=4). (**C**) The number of phagocytized red blood cells is shown relative to that of by *Apoe*$^{-/-}$ or *Apoe*$^{-/-}$*Rcan1*$^{-/-}$ macrophages (means±s.em; n=4). Student's t-test *p=0.011, **p<0.0077, ***p<0.0001.

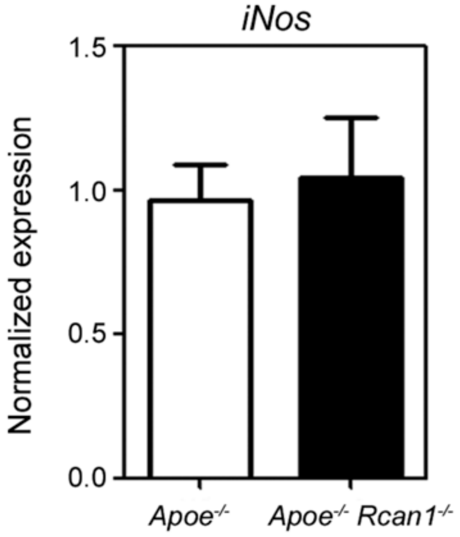

Figure S13. Rcan1 does not regulate *iNos* expression in atherosclerotic lesions. Quantitative PCR analysis of *iNos* mRNA expression in the aortic arch of atherosclerotic *Apoe*$^{-/-}$ (n=12) and *Apoe*$^{-/-}$*Rcan1*$^{-/-}$ mice (n=13) pooled from three independent experiments. mRNA amounts were normalized to *m36B4* expression (means±s.e.m).

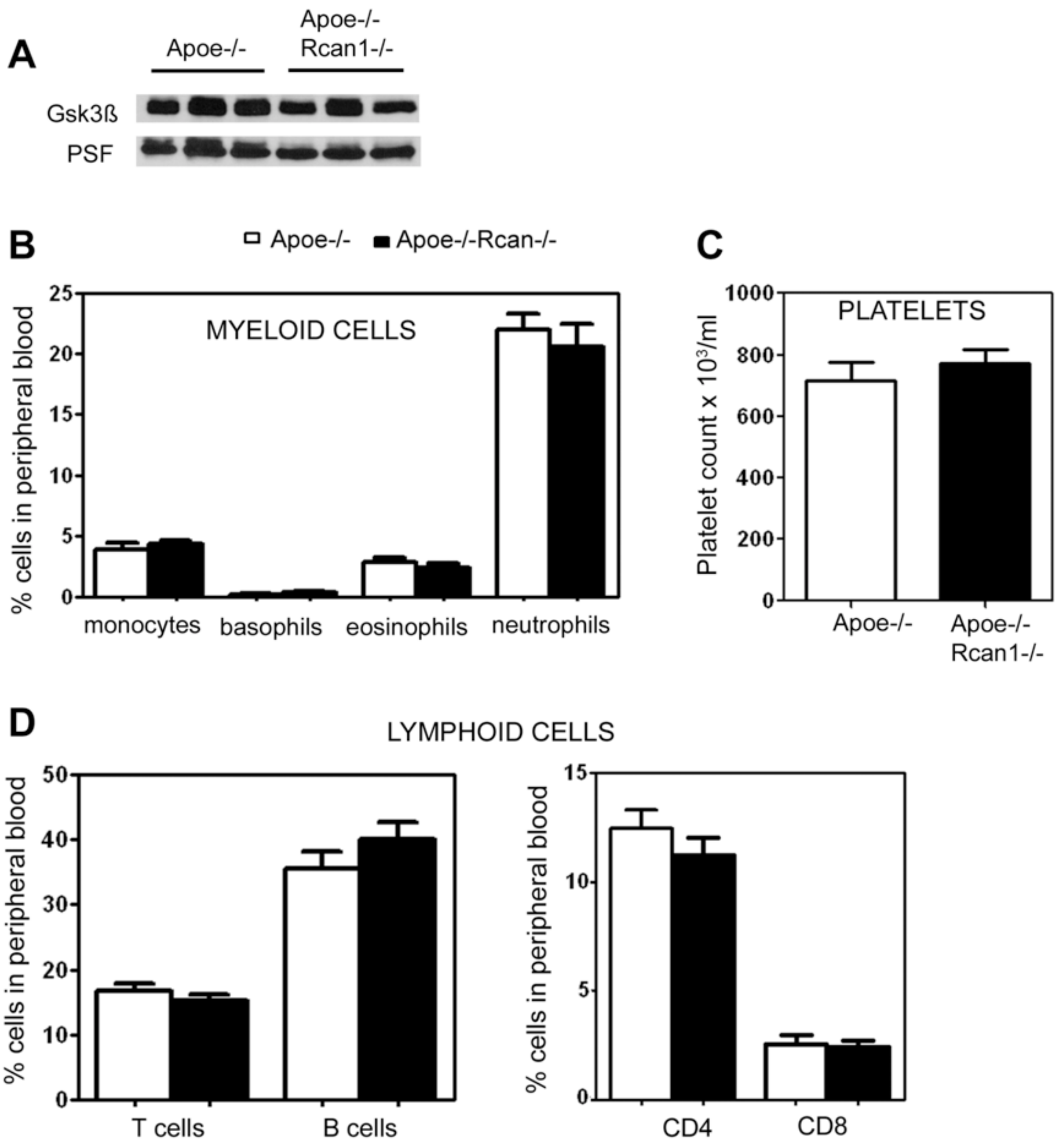

Figure S14. Rcan1 does not alter the nature of BM reconstitution. (**A**) Immunoblot analysis of Gsk3ß and PSF expression in BM cells of *Apoe*$^{-/-}$ mice transplanted with BM from *Apoe*$^{-/-}$ or *Apoe*$^{-/-}$*Rcan1*$^{-/-}$ mice. (**B - D**) Analysis of leukocyte populations 4 weeks after instilling *Apoe*$^{-/-}$ mice with BM from *Apoe*$^{-/-}$ (n=8) or *Apoe*$^{-/-}$*Rcan1*$^{-/-}$ mice (n=8). Data were pooled from two independent experiments and show (**B**) percentage of myeloid cell populations, (**C**) number of platelets and (**D**) percentage of lymphoid cell populations (means±s.em) and were pooled from two independent experiments.

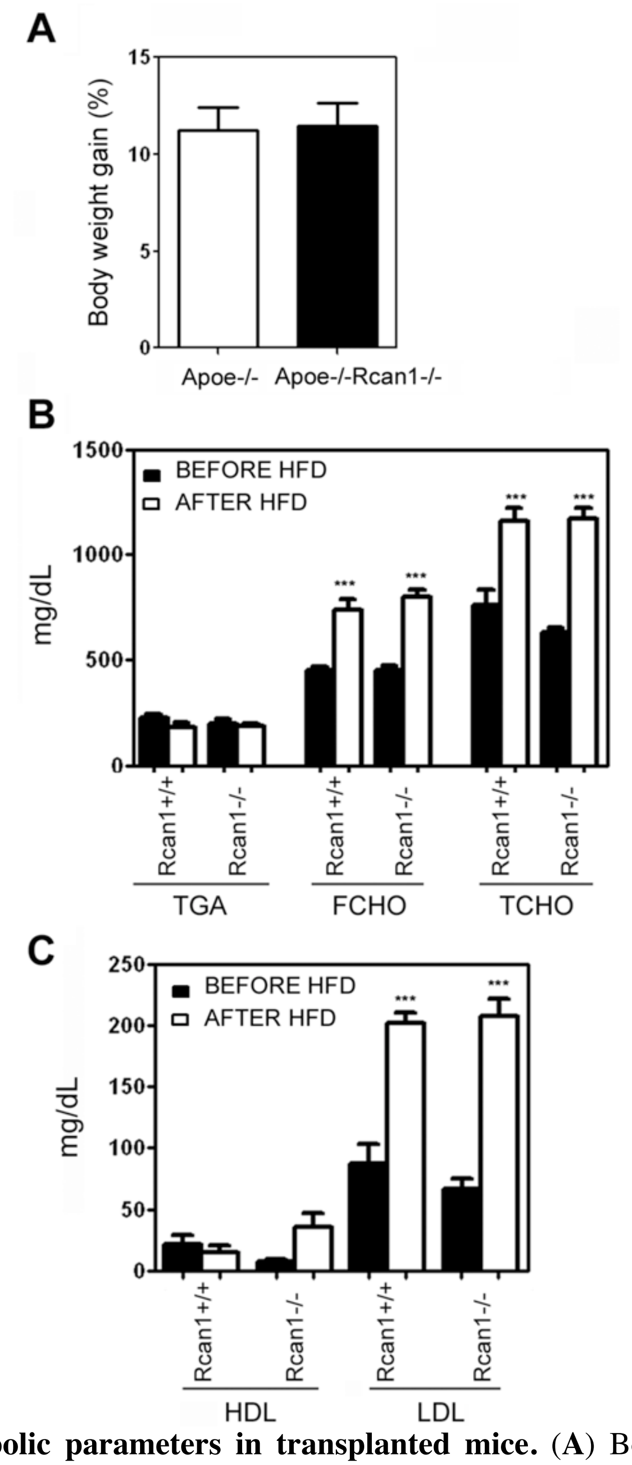

Figure S15. Metabolic parameters in transplanted mice. (**A**) Body weight gain of *Apoe*$^{-/-}$ mice transplanted with BM from *Apoe*$^{-/-}$ (Rcan1+/+; n=16) or *Apoe*$^{-/-}$*Rcan1*$^{-/-}$ mice (Rcan1-/-; n=15) after 6 weeks on a HFD. (**B**) Serum concentrations of triglyceride, total cholesterol (TCHO) and free cholesterol (FCHO) in these mice before and after the HFD (means±s.e.m). (**C**) Serum concentrations of HDL and LDL in the same mice (means±s.e.m). Data were pooled from three independent experiments. One-way ANOVA, ***p<0.0001.

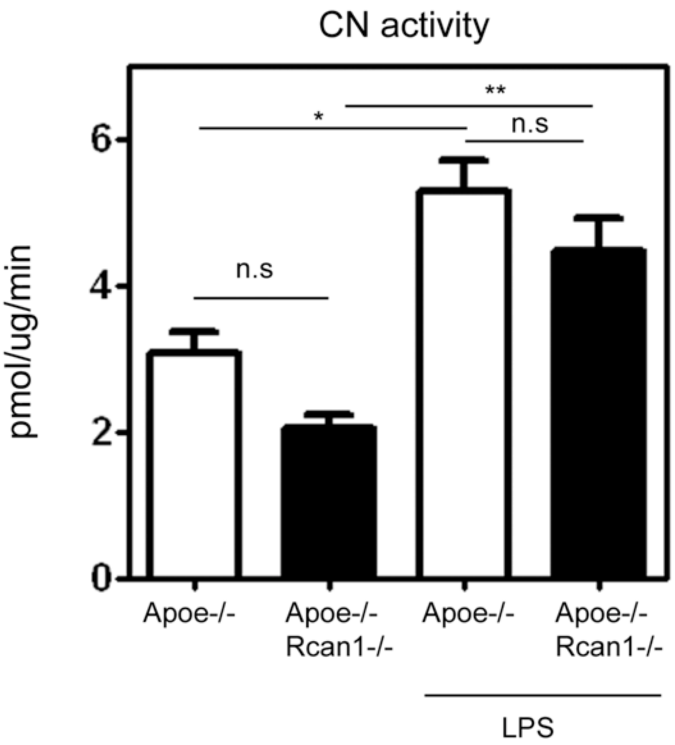

Figure S16. Rcan1 gene targeting does not alter CN activity in macrophages. CN enzymatic activity against phosphopeptide RII measured in *Apoe*$^{-/-}$ or *Apoe*$^{-/-}$*Rcan1*$^{-/-}$ peritoneal macrophages that were unstimulated or treated with 1μM lypopolysacharide (LPS) for 20 minutes. Values are the means ± SEM of duplicate determinations for each condition from four independent experiments. One-way ANOVA, *p=0.017, **p=0.004, n.s., non-significant.
